# Inter-rater Reliability of 4-Item Arterial Doppler Waveform Classification System for Description of Arterial Doppler Waveforms

**DOI:** 10.3389/fcvm.2020.584274

**Published:** 2020-10-26

**Authors:** Rui Zhao, Damien Lanéelle, Meiying Gao, Yuwei Fu, Yisha Tong, Robert Scissons, Chaoyang Wen, Guillaume Mahé

**Affiliations:** ^1^Peking University International Hospital, Haidian, China; ^2^INSERM U1075, Université de Caen Normandie, Caen, France; ^3^Vascular Medicine Department, Centre Hospitalier Universitaire de Caen, Caen, France; ^4^Department of Vascular Surgery, Austin Health, University of Melbourne, Melbourne, VIC, Australia; ^5^Jobst Vascular Institute, Toledo, OH, United States; ^6^Vascular Medicine Department, Centre Hospitalier Universitaire (CHU) de Rennes, Rennes, France; ^7^INSERM CIC1414 CIC Rennes, Rennes, France

**Keywords:** Doppler waveforms, peripheral artery disease, ultrasound, classification, reliability, Doppler (DP) spectrum

## Abstract

**Background:** Non-invasive Doppler waveform (DW) analysis is a widely adopted method for detecting and evaluating lower extremity peripheral artery disease (PAD). Previous investigations have reported that broad heterogeneity in the description of Doppler waveforms is reduced by using a classification method. The reliability of arterial Doppler classification, however, is unknown.

**Purpose:** The purpose of this study is to assess the reliability of a 4-category arterial DW classification method among Chinese sonographers.

**Methods:** During a national ultrasound conference in China attendees were invited to classify thirty arterial Doppler waveforms. After viewing a 4-category (triphasic, biphasic, monophasic, and other) arterial Doppler waveform descriptor presentation, attendees were asked to classify 15 continuous wave (CW) and 15 pulsed wave (PW) Doppler waveforms. Their responses were recorded via mobile phone and the reliability of this 4-category classification was estimated by Fleiss' Kappa inter-rater statistical analysis.

**Results:** One hundred and seventy-eight attendees participated in the analysis. The Kappa coefficient of Fleiss (κ) for all attendees was 0.522 (*p* < 0.005) with 95% confidence interval (CI): 0.520–0.523. The reliability of the waveform descriptor *triphasic* was the highest (κ = 0.621, *p* < 0.005), and *other* was the lowest (κ = 0.341, *p* < 0.005).

**Conclusion:** The inter-rater reliability of a 4-category arterial Doppler waveform classification by Chinese sonographers is considered weak (κ = 0.522, CI95%: 0.520–0.523, *p* < 0.005). This study reinforces the importance of assessing DW classification reliability and the development of DW descriptors that are more accurately predictive of clinical hemodynamic events.

## Background

Doppler ultrasound is routinely used for the non-invasive evaluation of lower extremity peripheral artery disease (PAD) and arterial Doppler waveform (DW) analysis provides a subjective method for categorizing arterial disease (1). In extremities at rest, Doppler waveforms are reflective of high resistive vascular beds. In the absence of PAD, arterial Doppler waveforms are multiphasic with high forward flow during systole, flow reversal in early diastole, and a smaller forward flow component in late diastole (2). As the severity of PAD increases the forward flow component in late diastole is lost and the DW progresses from a biphasic (two phase) to monophasic (single phase) waveform pattern (3). The absence of diastolic flow reversal is an important element in the non-invasive diagnosis of diseased blood vessels (4). For low-resistant vascular beds such as the brain, kidneys and liver, Doppler waveforms display continuous forward flow and the presence of atherosclerotic disease is assessed by changes in mean velocity and systolic acceleration ratio (5).

While arterial DW analysis is commonly used for the categorization of PAD severity (6), waveform definitions are not standardized and have contradictory characteristics for the same waveform descriptor in literature (7). These inconsistencies have perpetuated confusion in DW classification by vascular ultrasound professionals and other medical care providers (8). Studies have demonstrated there is significant heterogeneity in DW descriptions of physicians and residents, which can be significantly reduced with standardized waveform nomenclature (8, 9). A 4-category arterial DW classification (*triphasic, biphasic, monophasic*, and *others*) has previously been reported (10), however, the reliability of this classification has only been studied on small and heterogeneous population in the United States (US) and remains unknown in China. This study aims to assess the reliability of this classification among Chinese sonographers.

## Methods

This study was validated by the ethical committee of Rennes (France) and registered on clinicaltrials.gov (NCT03827512). During a 2019 national ultrasound congress in Beijing, attendees were invited to evaluate the reliability of a 4-category DW classification. Attendees were not selected and freely chose to answer the questionnaire. A DW survey previously developed and used in the US study was presented to attendees (10). The survey depicted thirty Doppler waveforms: 15 PW and 15 CW arterial Doppler waveforms (Supplementary Figure 1). Each CW and PW waveform group had 10 normal (triphasic or biphasic), 4 abnormal (monophasic) waveforms with varying levels of peripheral (outflow) resistance and 1 “other” waveform: a CW artery waveform with significant venous interference and a PW waveform within the neck of an arterial pseudoaneurysm.

### Study Design

The 4-category Doppler waveform classification system was presented in Chinese prior to the online questionnaire. The classification method used the following predefined waveform descriptors:

- Triphasic: three phases—forward flow, flow reversal, and a second forward component.
- Biphasic: two phases—one forward flow and one reverse component.
- Monophasic: single phase—forward flow with no reverse flow component.
- *Other*: waveforms considered neither triphasic, biphasic, nor monophasic or a waveform that could not be categorized.

After the waveform classification presentation made as on oral communication during the congress, an online questionnaire was provided. The questionnaire included general questions about their experience and the location where they practiced. The classification was displayed together with the questions to avoid a possible memory bias. DW images were of equal quality and remained on the conference presentation screen for 1 min each for a total of 30 min. All attendees proceeded to the next DW in the same time. Each Doppler waveform was numbered from 1 to 30 and attendees were invited to select one of the 4-category previously defined waveform descriptors via their mobile phone. The CW and PW waveforms were alternated and the responses entered into a database for correct answer comparison in accordance with the original presentation (10). Two answers were accepted as correct responses in four CW (waveform N°5, 13, 27, 15) and five PW (waveform N°6, 18, 20, 24, 28). “Other” was considered an incorrect response for all waveforms, excluding CW waveform N°29 and PW N°6.

### Statistical Analysis

Results are expressed as a median (first to third quartile) for quantitative variables and percentage for categorical variables. Attendee DW inter-rater reliability was calculated using the Kappa coefficient of Fleiss (κ) with corresponding 95% confidence intervals (11). We performed a comparison of the number of correct responses between the most and least experienced group of attendees, defined by the median age of experience. We also performed a complementary analysis of reliability by merging the 2 sub-categories “triphasic” and “biphasic” that can be considered as “normal category.” Two different interpretations of Kappa values were used: (1) Landis and Koch and (2) McHugh (12, 13), the latter is considered a more stringent methodology. While the Landis and Koch use: 0.21–0.40: fair; 0.41–0.60: moderate, 0.61–0.80: substantial; >0.80: almost perfect reliability, the McHugh method uses: 0.21–0.39: minimal; 0.40–0.59: weak; 0.60–0.79: moderate; 0.80–0.90: strong; >0.90: almost perfect.

The analysis was performed using R software version 3.0.1 (R Foundation for Statistical Computing, Vienna, Austria; www.R-project.org), with the package “irr” (14). Probability and coefficients were expressed using a 95% confidence interval; correct responses (in percent) were compared using the Chi-squared test. The *p*-value of <0.005 was considered statistically significant.

## Results

One hundred seventy-eight attendees with 1 to 33 years of ultrasound vascular experience and representing 23 Chinese provinces were included in the study. The median number of years working experience in vascular ultrasound was 7 years (first quartile–third quartile: 3–13 years).

### Correct Responses

The designated correct CW and PW responses and the average percentage of triphasic, biphasic, monophasic, and “other” responses by all attendees are presented in Table 1. The mean average of correctly identified waveforms was 74% (range 10–98%). The maximum value was 98% for CW waveform N°3 and PW waveform N°6. The minimal values were 10 % for CW waveform N°25 and 20% for PW waveform N°12. No statistical differences were noted between averages for all PW and CW Doppler waveforms (*p* > 0.005). There was no significant difference in correct responses between the group with more than 7 years of experience and the group with 7 years of experience or less (Table 2).

**Table 1:** Conference attendee response summary of 15 Continuous-Wave (CW) and 15 Pulsed-Wave (PW) arterial Doppler waveforms.

	**Triphasic**	**Biphasic**	**Monophasic**	**Other**	**Percentage of correct responses**
**CW waveform**					
1	0	15	**137**	26	77%
3	**175**	2	0	1	98%
5	**138**	**25**	12	3	92%
7	3	6	**165**	4	93%
9	15	**159**	1	3	89%
11	2	**166**	1	9	93%
13	**53**	**5**	0	120	33%
15	12	**151**	**7**	8	89%
17	0	2	**115**	61	65%
19	6	**154**	2	16	87%
21	8	**33**	124	13	19%
23	0	3	**156**	19	88%
25	2	**17**	145	14	10%
27	**134**	**26**	1	17	90%
29	1	3	1	**173**	97%
**PW waveform**					
2	3	39	**131**	5	74%
4	**163**	1	0	14	92%
6	2	99	2	**75**	98%
8	**141**	16	8	13	79%
10	1	20	**145**	12	81%
12	10	**36**	129	3	20%
14	43	**84**	4	47	47%
16	11	**163**	0	4	92%
18	**46**	**72**	5	55	66%
20	**150**	**22**	1	5	97%
22	3	**134**	31	10	75%
24	9	**135**	**17**	17	85%
26	4	34	**113**	27	63%
28	4	**35**	**136**	3	96%
30	0	31	**77**	70	43%

*Acceptable correct answers are in bold*.

**Table 2:** Comparison of interpretations of Doppler waveforms with 4-item classification according to experience.

**Doppler waveforms**	**Percentage of correct answers**		***p-*value**
	**experience of 1-7 years**	**experience of more than 7 years**	
**CW waveform**			
1	78%	76%	0.795
3	97%	100%	0.252
5	91%	92%	0.822
7	91%	94%	0.411
9	91%	88%	0.435
11	93%	93%	0.968
13	38%	27%	0.135
15	88%	82%	0.268
17	63%	66%	0.719
19	90%	83%	0.169
21	22%	15%	0.201
23	87%	89%	0.690
25	9%	10%	0.761
27	91%	89%	0.584
29	98%	97%	0.980
**PW waveform**			
2	72%	75%	0.674
4	90%	93%	0.445
6	98%	98%	0.982
8	76%	83%	0.224
10	82%	81%	0.791
12	20%	20%	0.940
14	49%	45%	0.646
16	92%	91%	0.753
18	66%	67%	0.833
20	96%	98%	0.699
22	73%	77%	0.542
24	83%	88%	0.431
26	63%	64%	0.967
28	20%	19%	0.909
30	38%	49%	0.136

### Reliability of 4-Category Arterial Doppler Classification

The Kappa coefficient of Fleiss for 178 attendees was 0.522 (CI95%: 0.520–0.523, *p* < 0.005). The inter-rater reliability for classifications in each category is presented in Table 3.

**Table 3:** Conference attendee inter rater reliability summary of 4-category Doppler waveform classification system.

**Category**	**Fleiss' Kappa**	***P*-value**
Monophasic	0.591	<0.005
Biphasic	0.481	<0.005
Triphasic	0.631	<0.005
Other	0.341	<0.005
**Global Doppler classification**	**0.522**	**<0.005**

*Overall reliability of the classification in bold*.

### Reliability of 3-Category Arterial Doppler Classification

A similar inter-rater reliability was found when we merged together the “*triphasic*” and “*biphasic*” categories with, however, a reliability for this new category of κ = 0.559 (CI95%: 0.557–0.561, *p* < 0.005), results are presented in Table 4.

**Table 4:** Conference attendee inter rater reliability of 4-category Doppler waveform classification system with a merging of the triphasic and biphasic categories.

**Category**	**Fleiss' Kappa**	***P*-value**
Monophasic	0.591	<0.005
Biphasic and triphasic	0.559	<0.005
Other	0.341	<0.005
**Global Doppler classification**	**0.522**	** <0.005**

*Overall reliability of the classification in bold*.

## Discussion

In our investigation, the 4-category classification system produced “moderate” or “weak” inter-rater interpretation reliability (κ = 0.522, CI95%: 0.520–0.523, *p* < 0.005). Previous investigations have demonstrated the importance of having a definitive classification system to reduce the heterogeneity of DW descriptions (8–10). To prevent DW characterization from being excessively discriminatory the classification system should not be too restrictive, e.g., normal and abnormal (two categories). Conversely, a classification system with an excessive number of DW descriptors might create confusion and the potential for DW characterization overlapping into more than one category.

The “moderate” or “weak” inter-rater reliability in our study was surprising. While we believe the more stringent guideline as offered by McHugh may be more appropriate for clinical data or healthcare research, we believe a 4-category system produces greater agreement. There was “substantial” or “moderate” agreement with the triphasic descriptor (κ = 0.631, CI95%: 0.629–0.632) and slightly less (κ = 0.591, CI95%: 0.589–0.593) using the monophasic term. The biphasic waveform descriptor, however, was less reliable (κ = 0.481, CI95%: 0.479–0.482) and “fair” or “minimal” reliability (κ = 0.341, CI95%: 0.338–0.344) when using “Other” suggests this waveform descriptor term should be avoided.

The inter-observer reliability noted in this study should be weighed with consideration that congress attendees were using the 4-category classification system for the first time. Improved reliability should be expected when sonographers became more familiar with the 4-category classification method, however, this assumption remains to be validated. Additionally, the waveform classification presentation exhibited to the attendees was made with schematic Doppler waveforms. It is likely that inter-observer reliability would improve if actual clinical waveforms were used instead of a pedagogical presentation.

While other waveform classification methods have been proposed: *Cathignol et Descotes* (5-categories) and Saint-Bonnet (12-categories) (3, 15), their interpretive reliability has also not been evaluated.

Of particular interest is the percentage of correct DW responses in this study. Conference attendees in China correctly identified 74% of the presented waveforms, which is remarkably similar (73%) to a previous study published in 2008 in the United States (10).

Comparisons in terms of reliability is difficult between the American study and the present Chinese study because the American population was smaller (*n* = 97) and included three different categories of healthcare professionals (students; physicians with an average à 8 years of experience and sonographers with an average of 15 years of experience). Moreover, the initial study did not include an analysis of reliability with a kappa value but simply a count of the correct answers. An additional analysis was performed to obtain an objective kappa value (16). The reliability of the overall population was poorer (κ = 0.378). Reliabilities for the 25 students without experience, for the 23 physicians with an average of 8 years (±5) of experience and for the 24 sonographers with an average of 15 years (±8) of experience were κ = 0.319, κ = 0.329, and κ = 0.462, respectively. If we only compare the reliability of the American sonographer subgroup (κ = 0.462) with that of our study (κ = 0.522), we observe that it is quite similar.

Another study among French vascular medicine residents using the same arterial DW questionnaire found a higher percentage of correct responses (82%) but with a small sample size. Of interest the reliability of the classification was not evaluated in this study due to a too small sample size (*n* = 19) (8). The high rate of good response can be explained by the fact that vascular medicine residents were familiar with the Doppler waveform analysis during the initial vascular medicine training. The strength of our study compared to previous work is to evaluate the reliability of this classification over a large and homogenous sample in terms of occupation and experience. A first American consensus about Doppler waveform interpretation has just been released in July 2020 to try to clarify the way of reporting Doppler waveforms in clinical practice but it did not address the reliability of Doppler waveform analysis (17).

## Study Limitations

The primary limitation of this study is that attendees from different parts of China were unfamiliar with the presented 4-category DW classification method. Additionally, the attendee presentation used a mixture of CW and PW waveforms from the original 2008 investigation (10). Second, in the absence of reliability calculations in previous studies, we did not calculate the number of subjects required. This is a relative limitation of this survey but the present study can be considered as a pilot study.

Third, Doppler waveforms were also presented without medical context, patient age of and the arterial bed being examined. Fourth, we did not investigate how DW classification can changed patient's management. The impact of arterial DW on clinical management remains an issue. We believe a reliable waveform classification system should incorporate the medical context and vascular bed being examined. Fifth, we did not assess the attendees' knowledge of the classification prior to the presentation. However, to our knowledge, this classification is not taught in China. Finally, while the estimation of intra-observer reliability was not achieved this was not the primary purpose of this study.

## Conclusion

The inter-rater reliability of a previous reported 4-category arterial Doppler waveform classification method among Chinese sonographers was considered weak (κ = 0.522, *p* < 0.005). This study reinforces the importance of assessing DW categorization reliability and the development of DW descriptors that are more accurately predictive of clinical hemodynamic events.

## Data Availability Statement

The raw data supporting the conclusions of this article will be made available by the authors, without undue reservation.

## Ethics Statement

This study was validated by the ethical committee of Rennes (France).

## Author Contributions

DL: literature search, data analysis, data interpretation, writing the report, and final approval of the manuscript. CW, YF, and MG: literature search, data collection, data analysis, data interpretation, revising the intellectual content, final approval of the manuscript, and redaction. RZ, YT, and RS: literature search, data collection, data analysis, data interpretation, revising the intellectual content, and final approval of the manuscript. GM: literature search, data collection, data analysis, data interpretation, writing the report, and final approval of the manuscript. All authors contributed to the article and approved the submitted version.

## Conflict of Interest

The authors declare that the research was conducted in the absence of any commercial or financial relationships that could be construed as a potential conflict of interest.
