# Supplementary material for: Inter-rater Reliability of 4-Item Arterial Doppler Waveform Classification System for Description of Arterial Doppler Waveforms
Source: Front Cardiovasc Med. 2020 Oct 26;7:584274. doi: 10.3389/fcvm.2020.584274 (PMC7649166; doi:10.3389/fcvm.2020.584274)
Supplement: Supplementary file 1 [file Presentation_1.PPTX]

## Slide 1
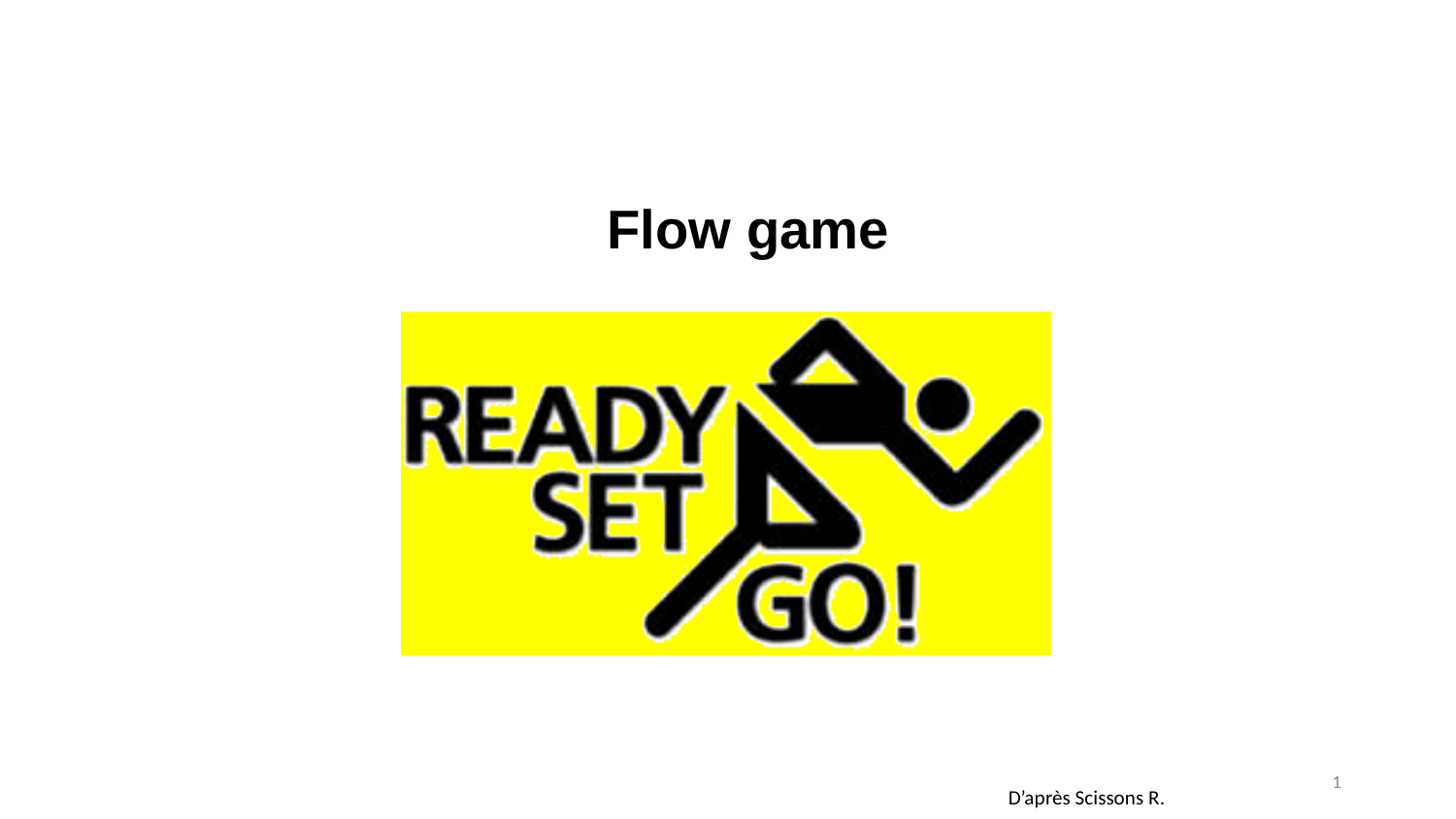

Flow game
1
D’après Scissons R.

## Slide 2
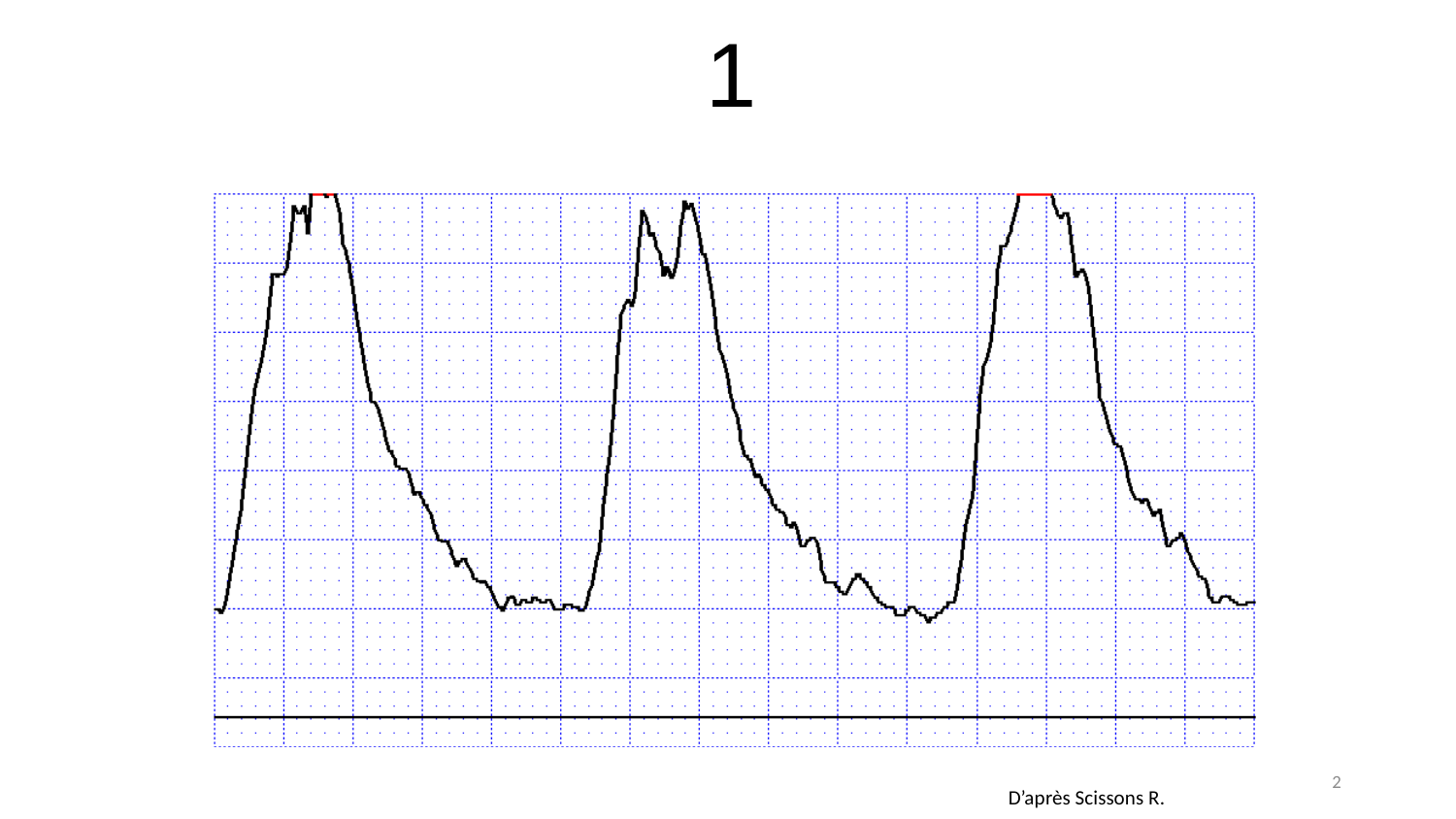

1
2
D’après Scissons R.

## Slide 3
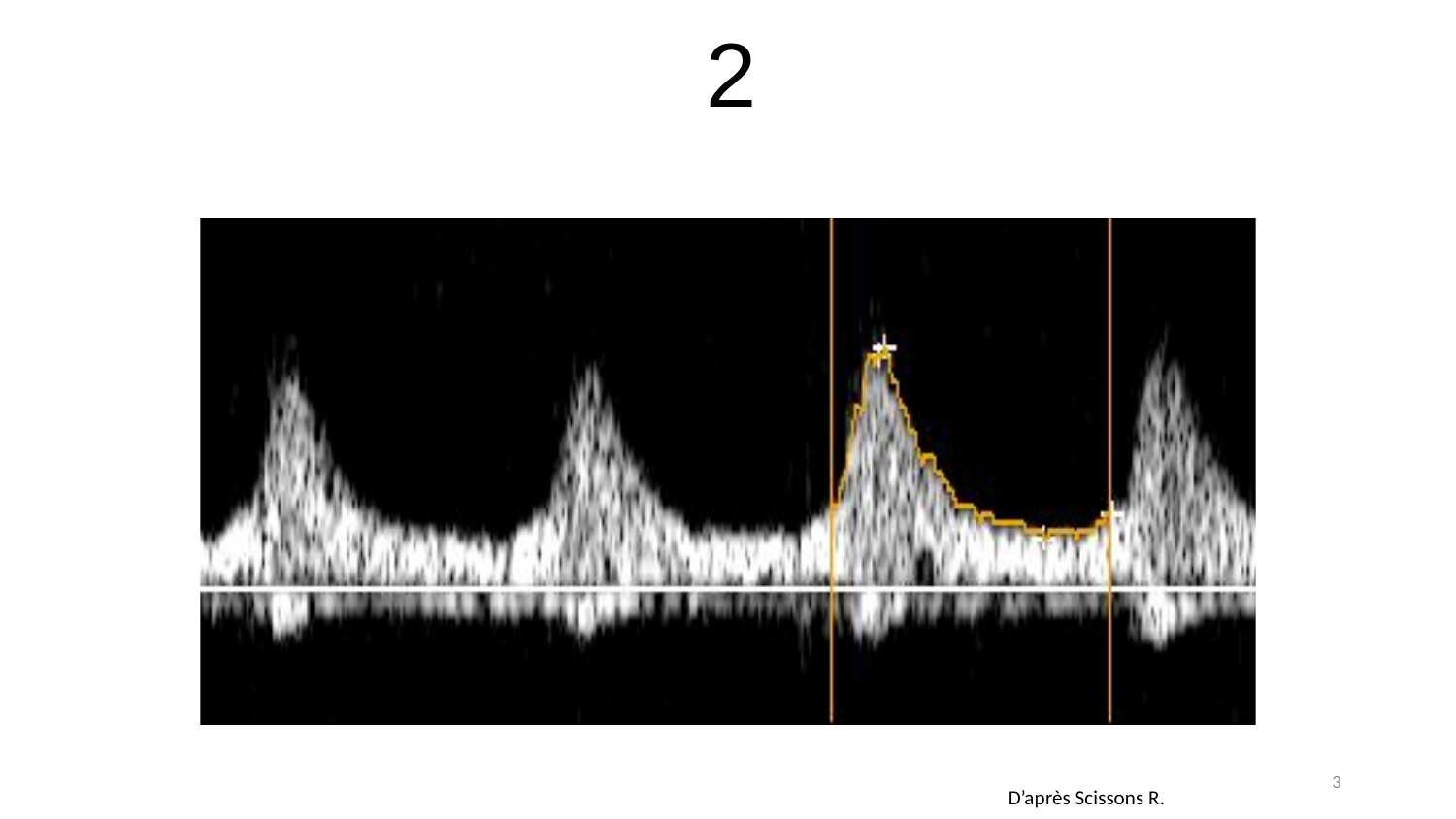

2
3
D’après Scissons R.

## Slide 4
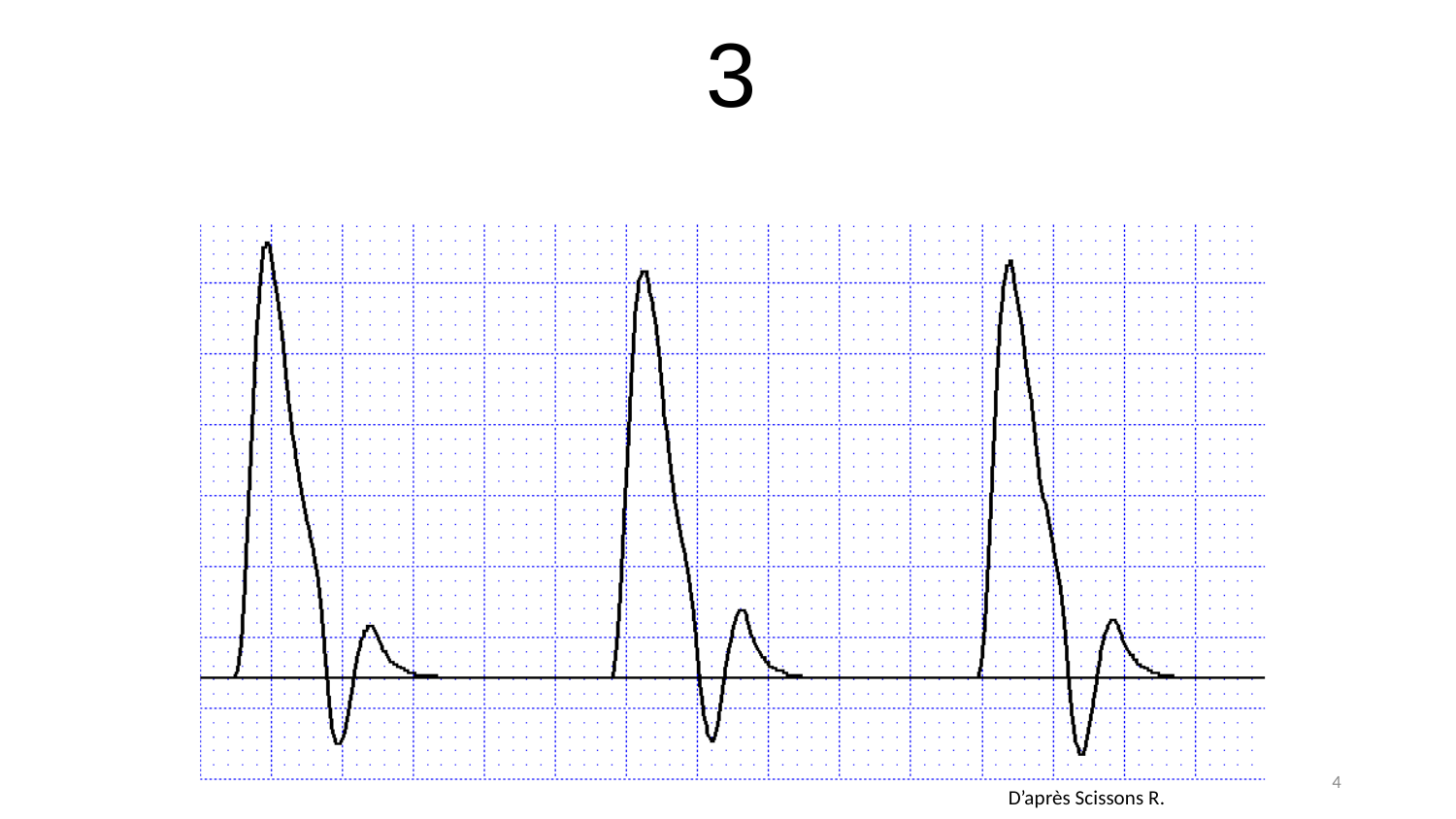

3
4
D’après Scissons R.

## Slide 5
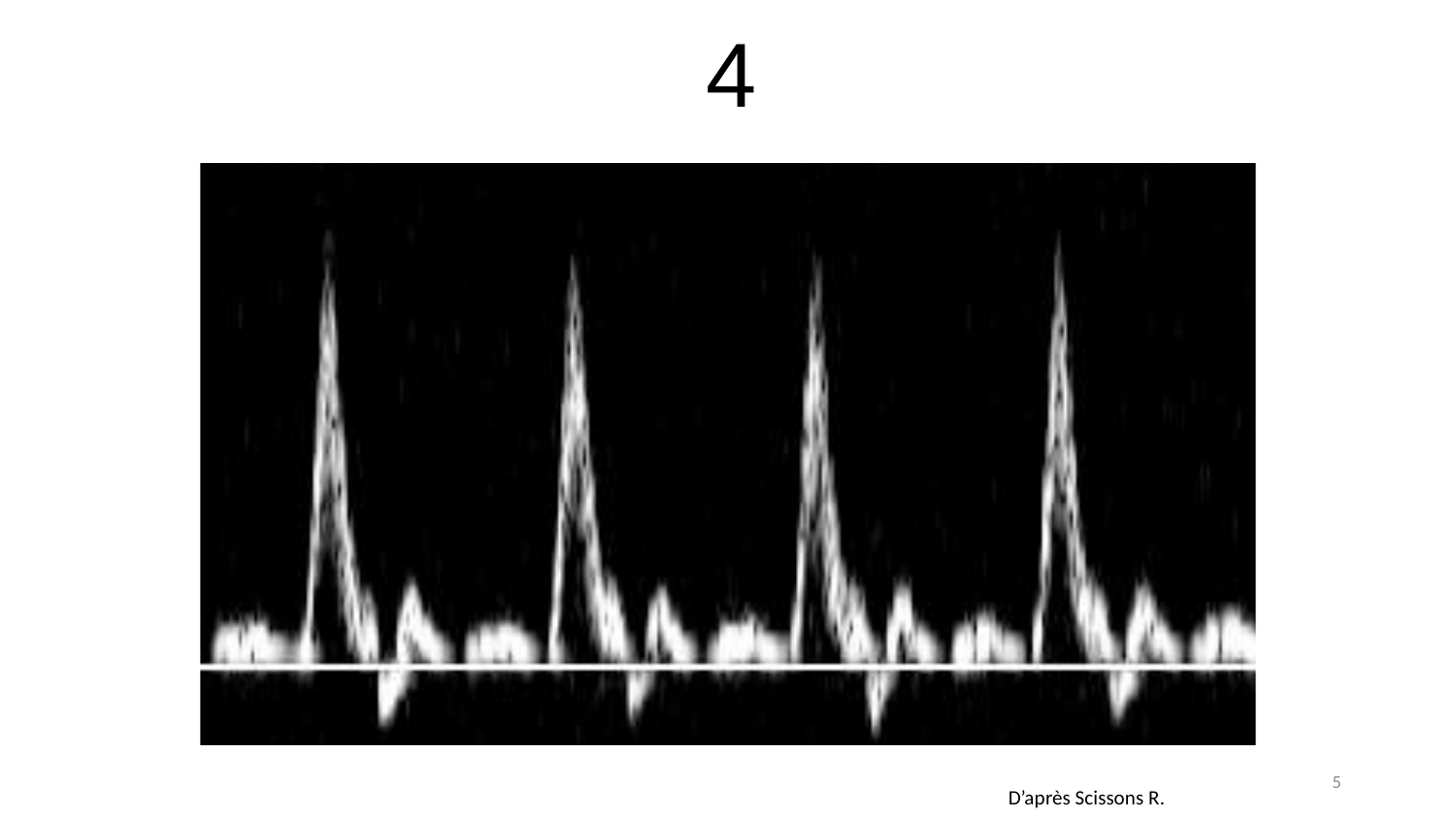

4
5
D’après Scissons R.

## Slide 6
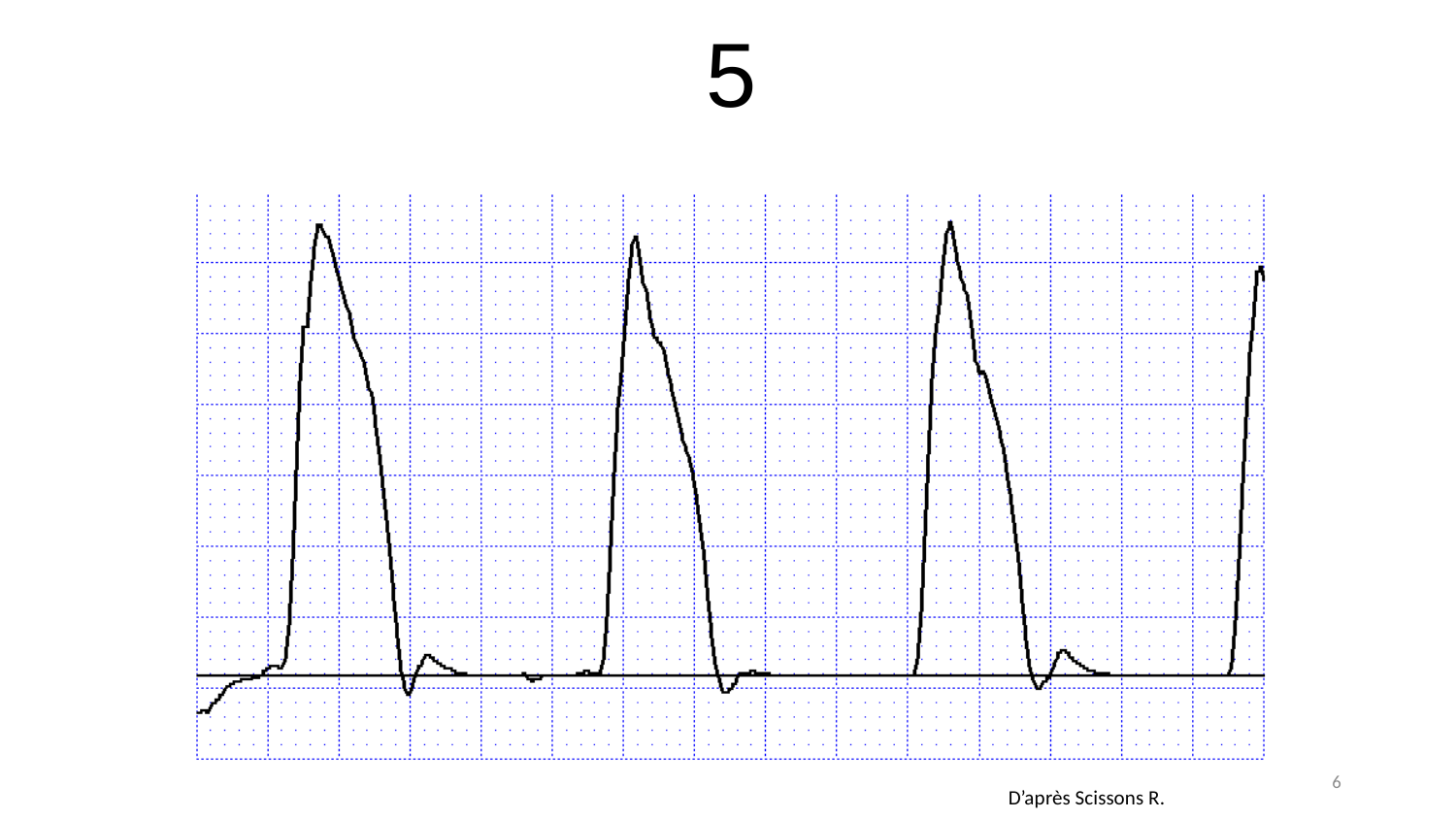

5
6
D’après Scissons R.

## Slide 7
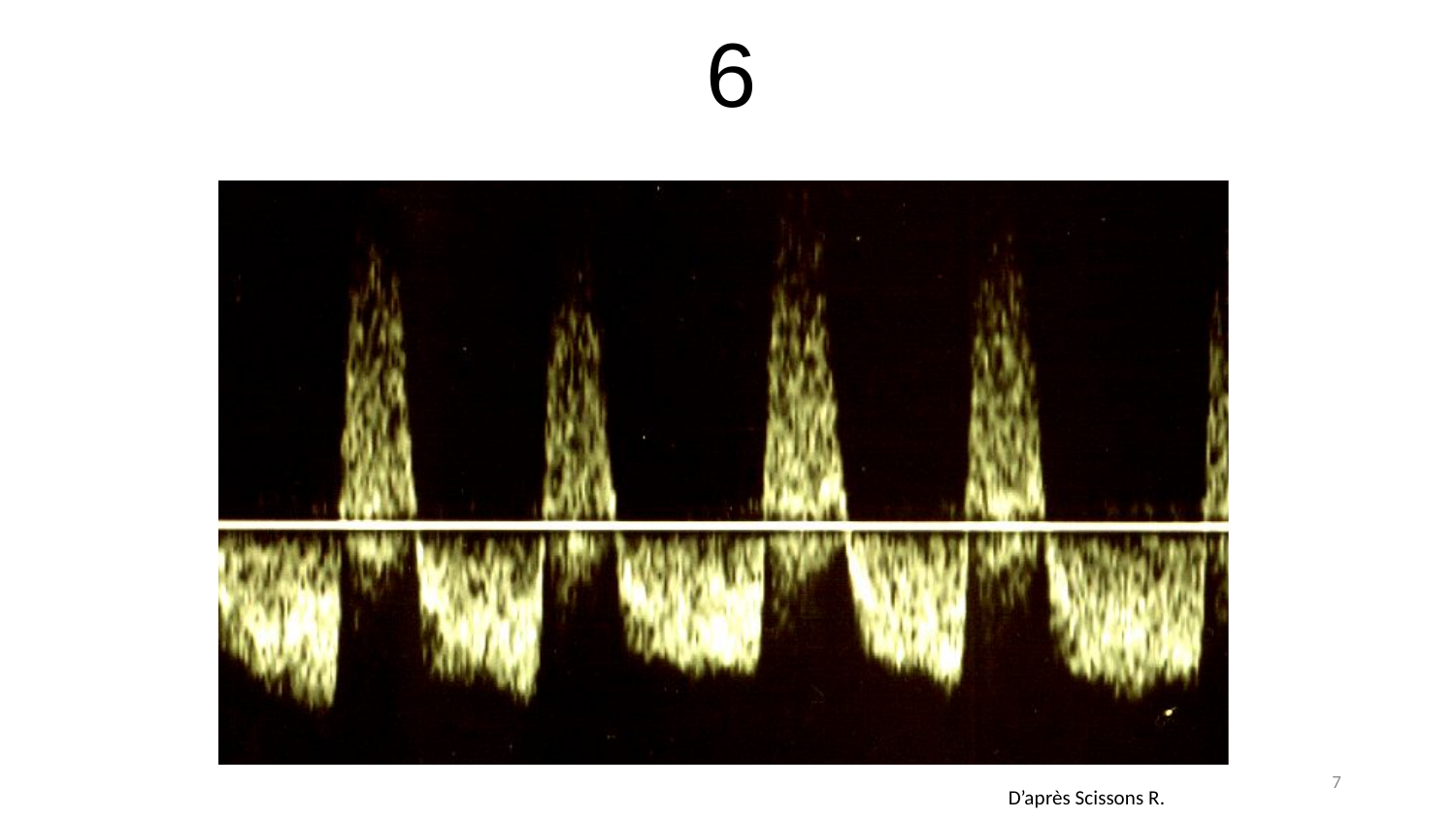

6
7
D’après Scissons R.

## Slide 8
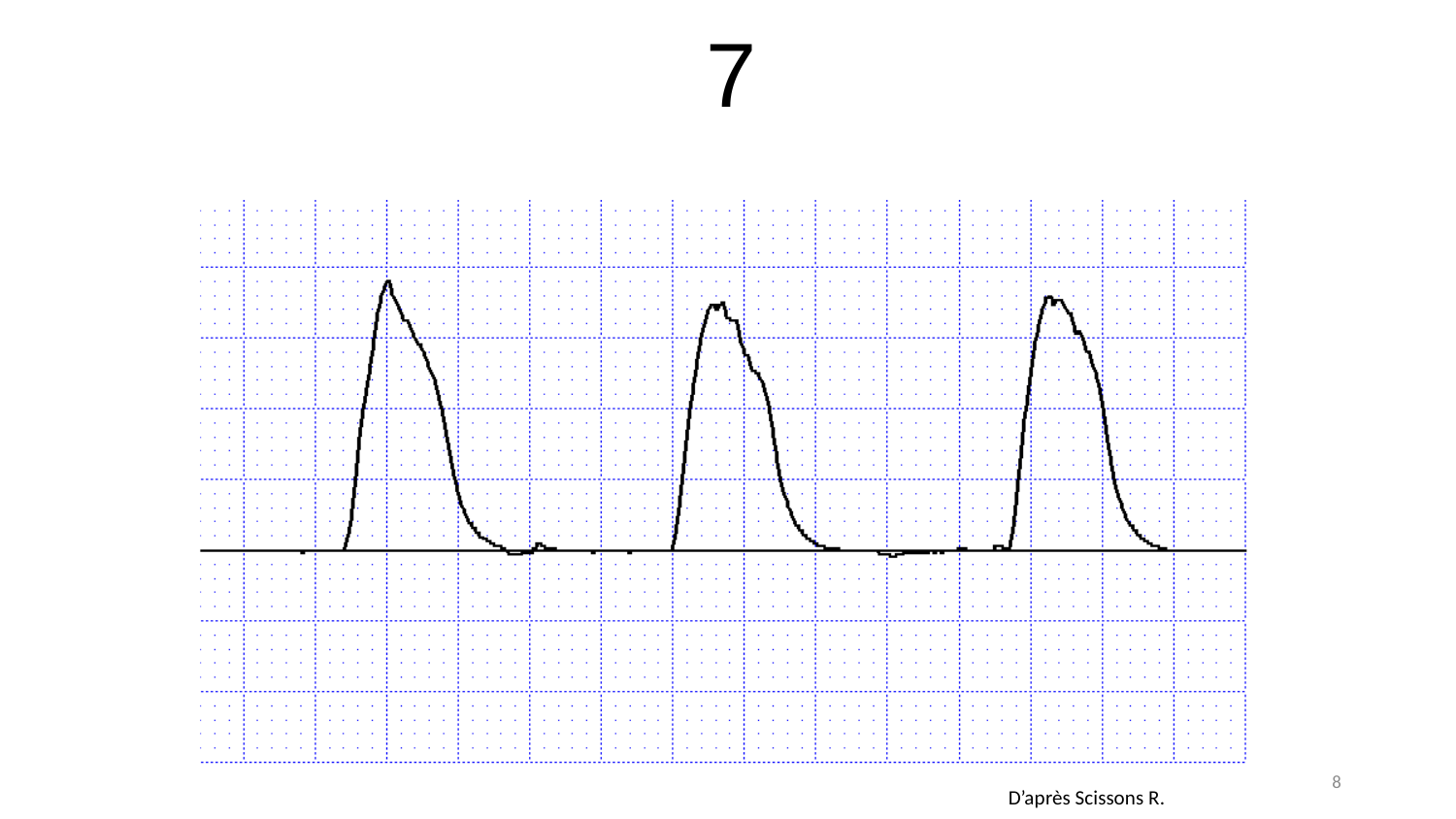

7
8
D’après Scissons R.

## Slide 9
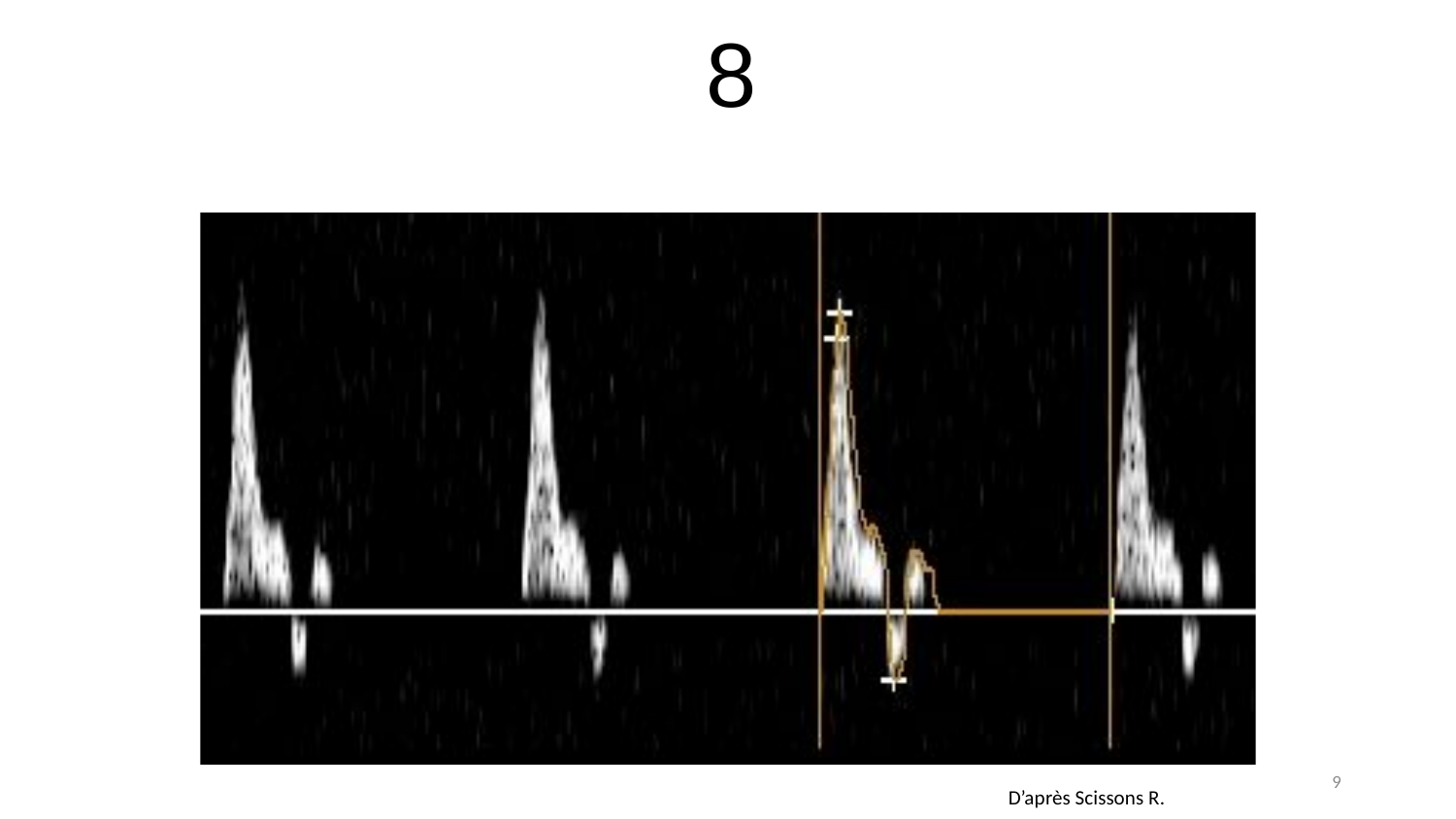

8
9
D’après Scissons R.

## Slide 10
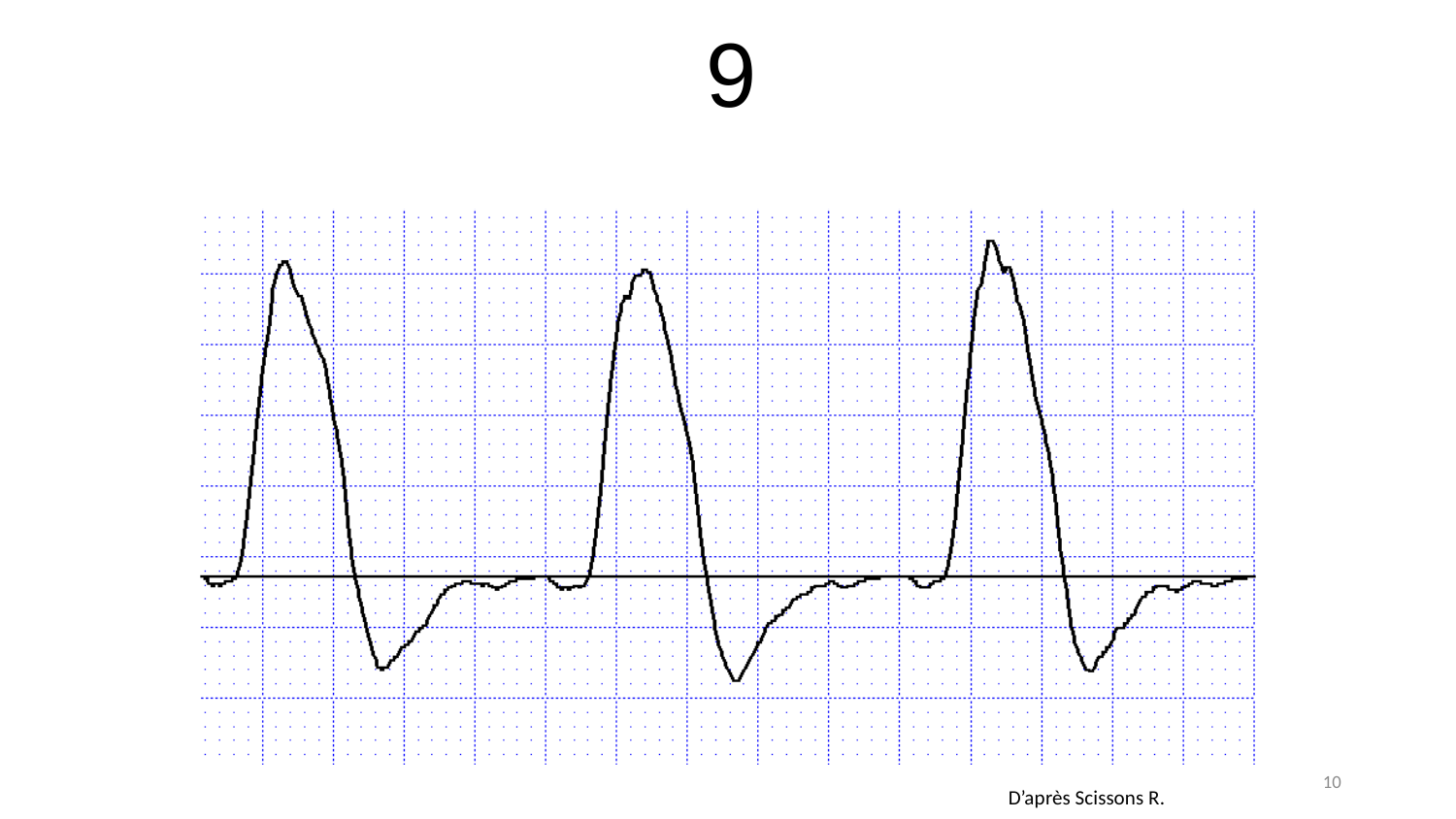

9
10
D’après Scissons R.

## Slide 11
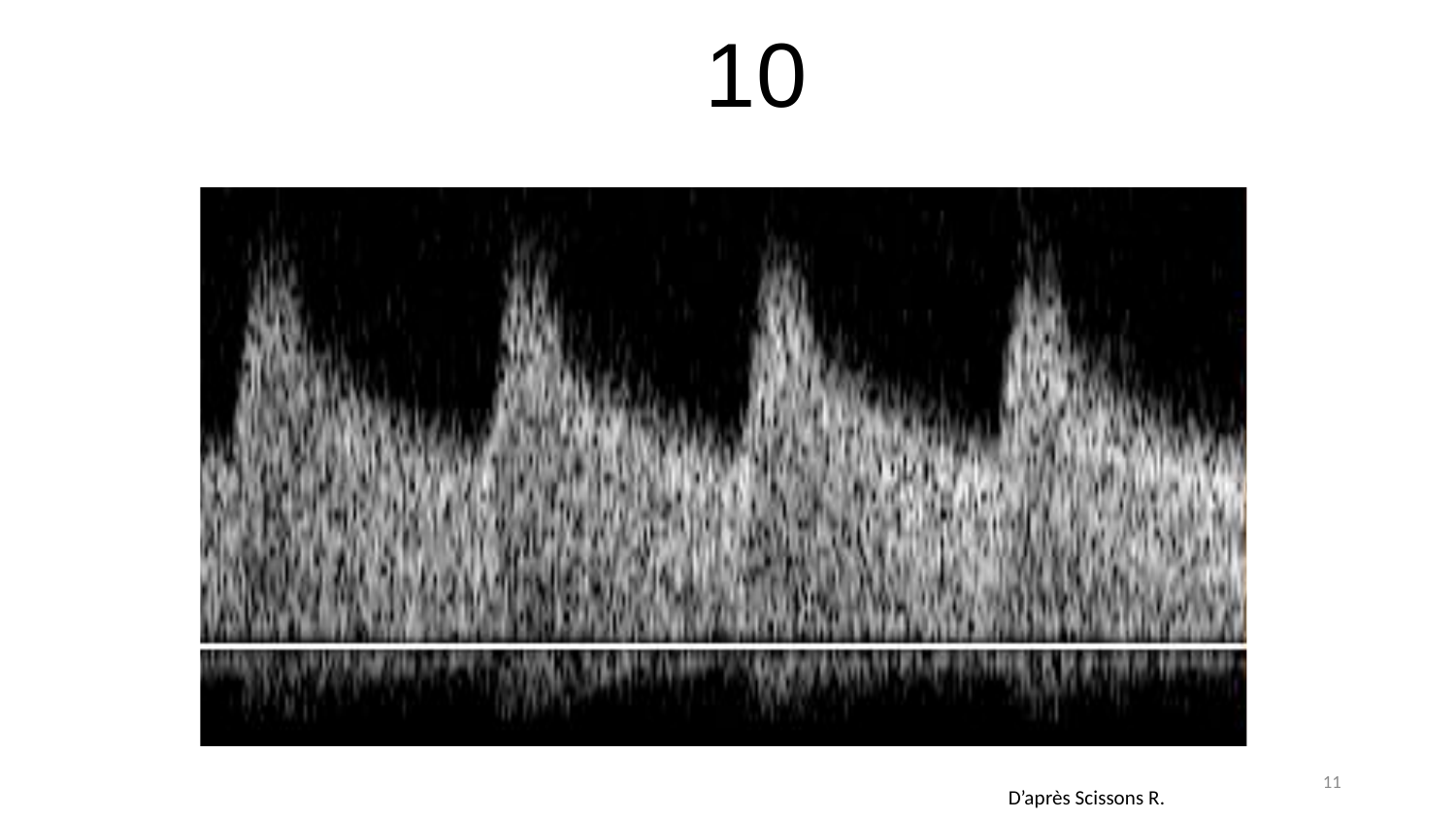

10
11
D’après Scissons R.

## Slide 12
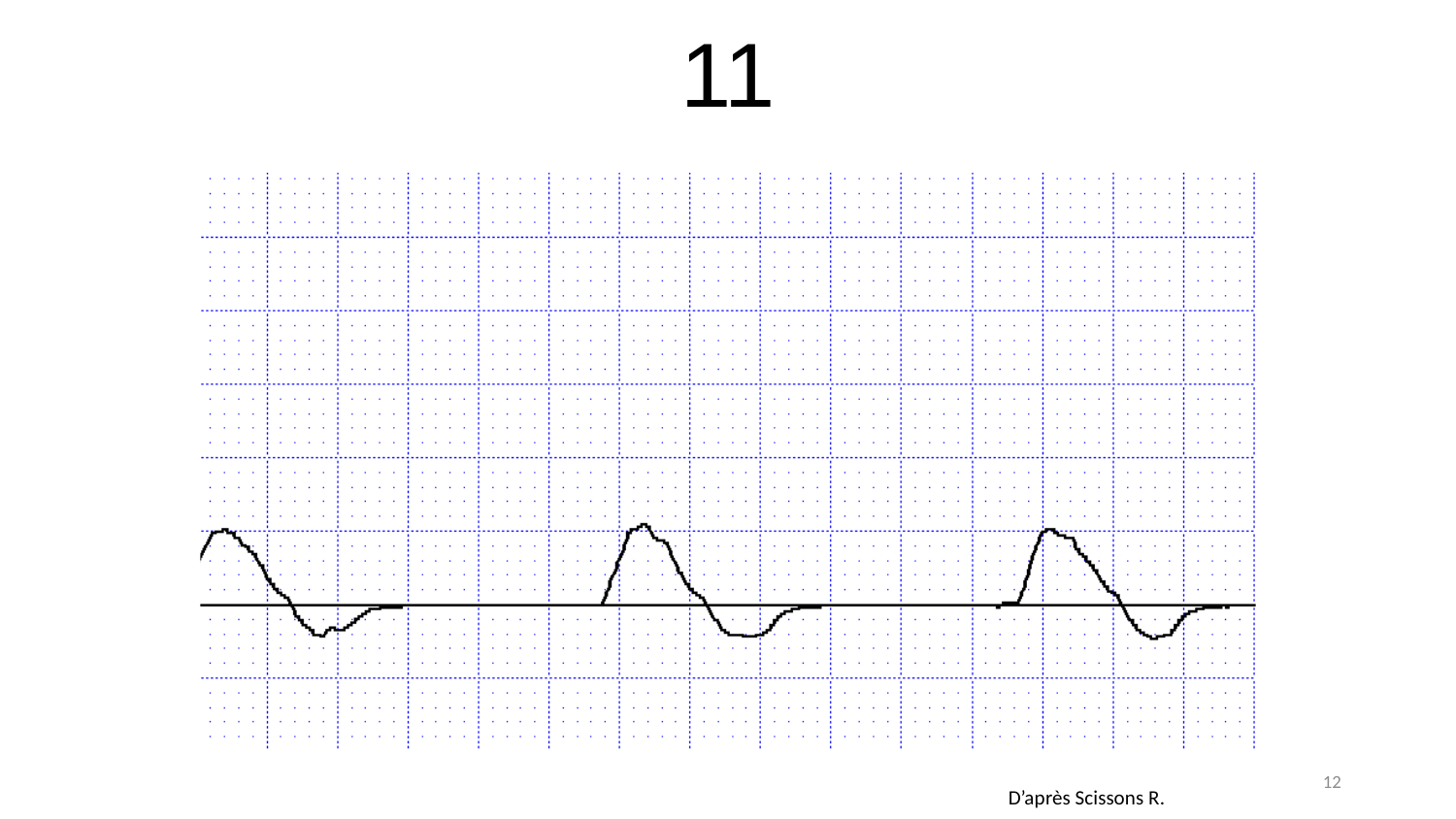

11
12
D’après Scissons R.

## Slide 13
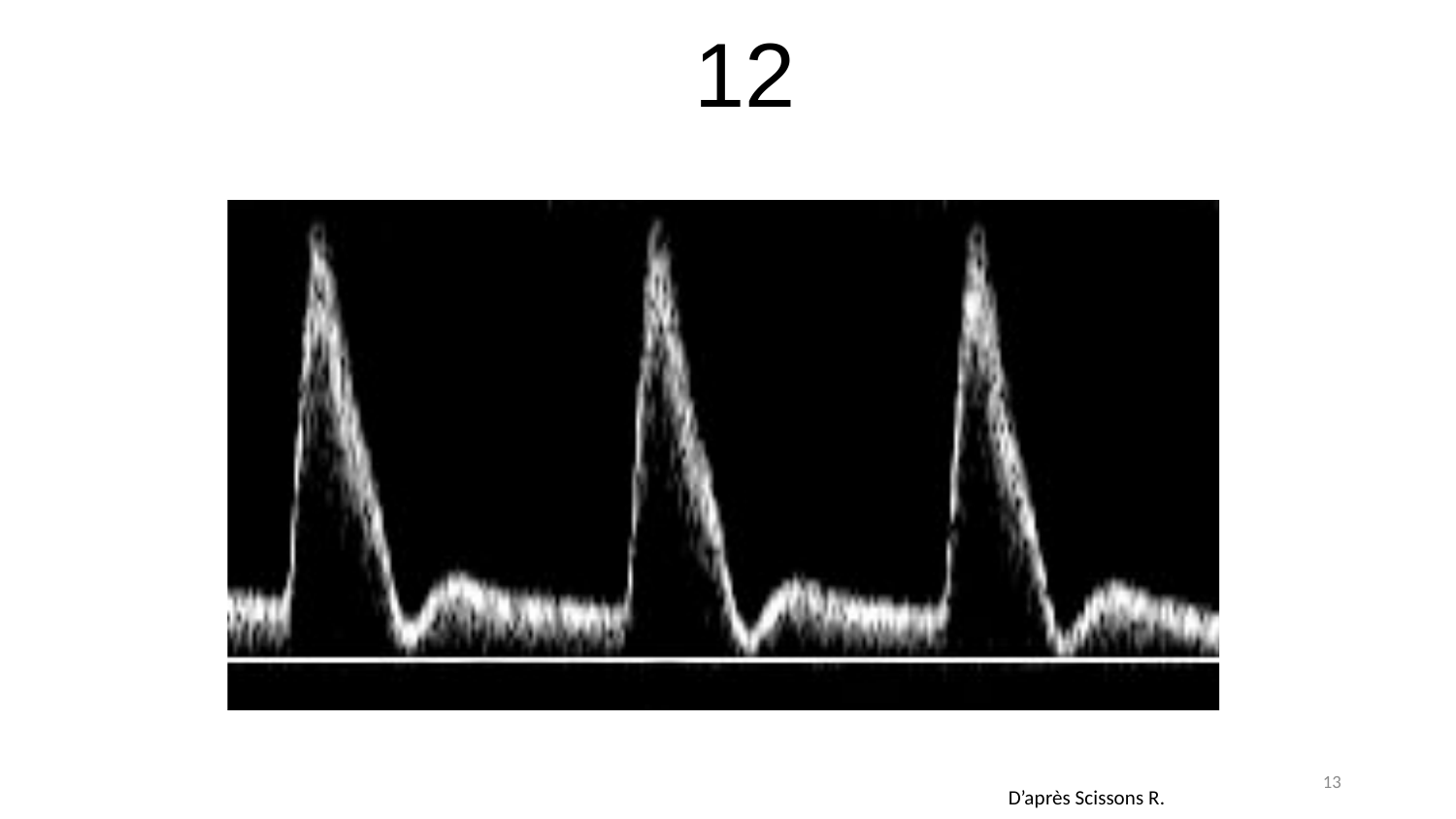

12
13
D’après Scissons R.

## Slide 14
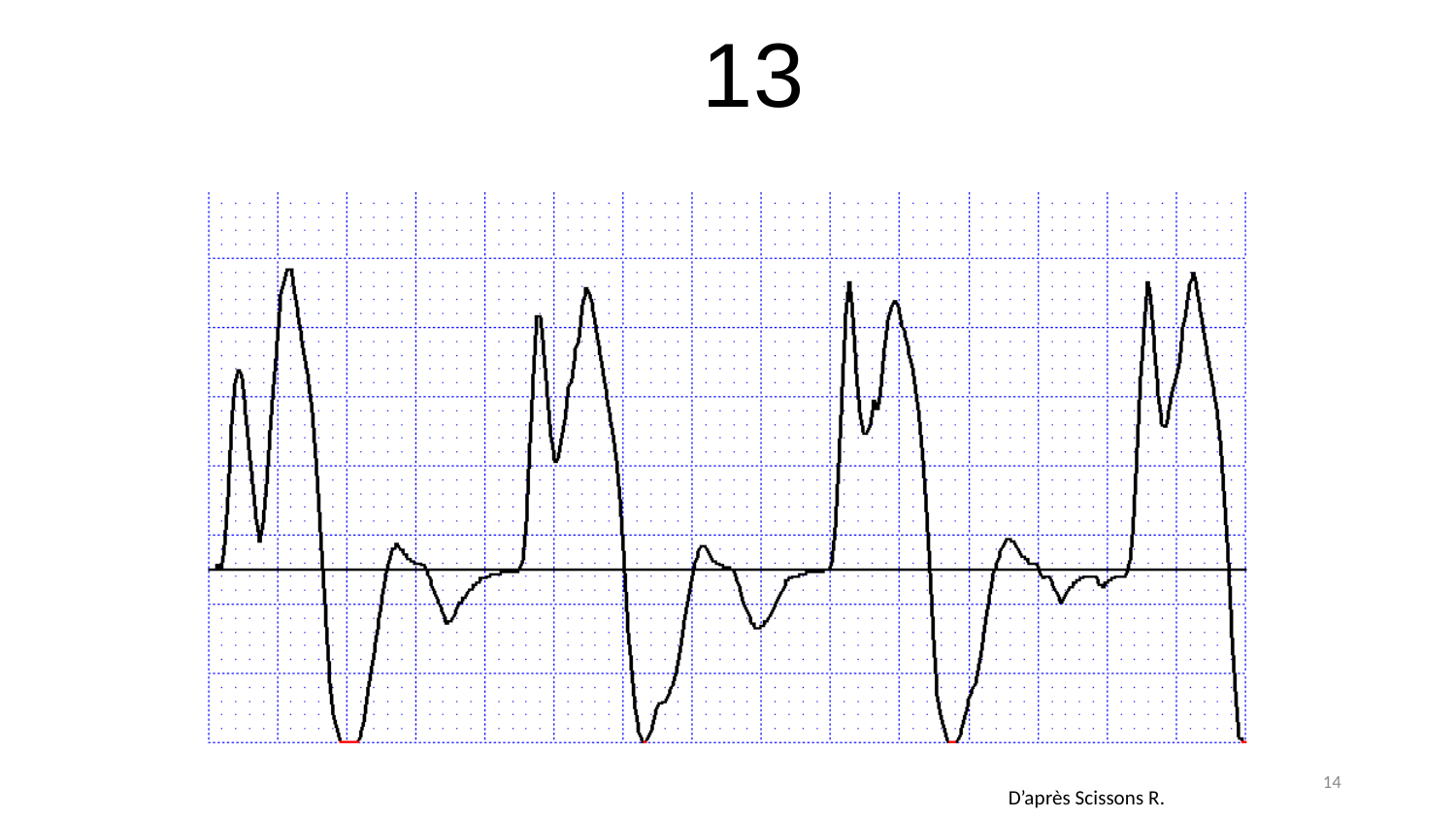

13
14
D’après Scissons R.

## Slide 15
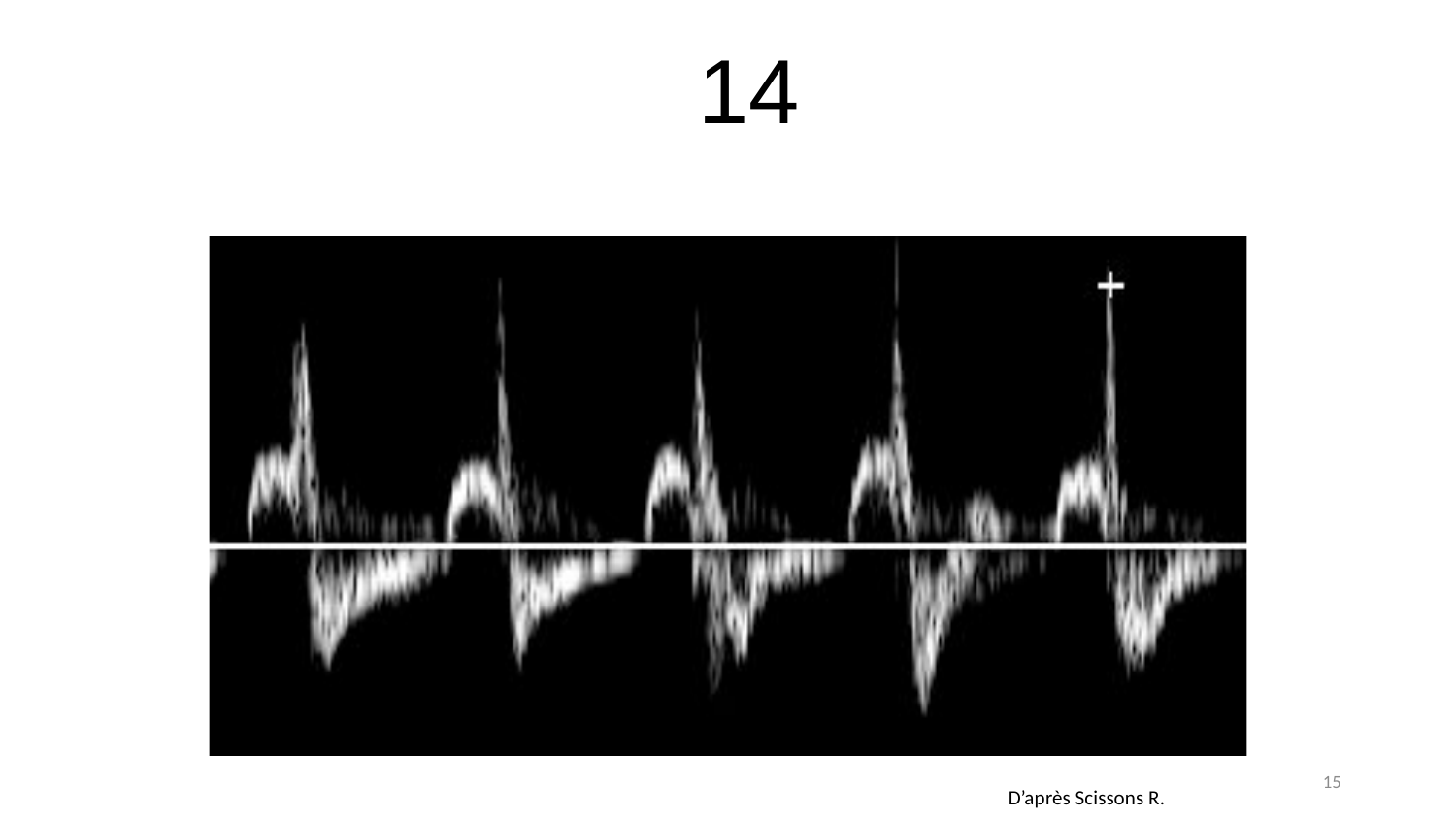

14
15
D’après Scissons R.

## Slide 16
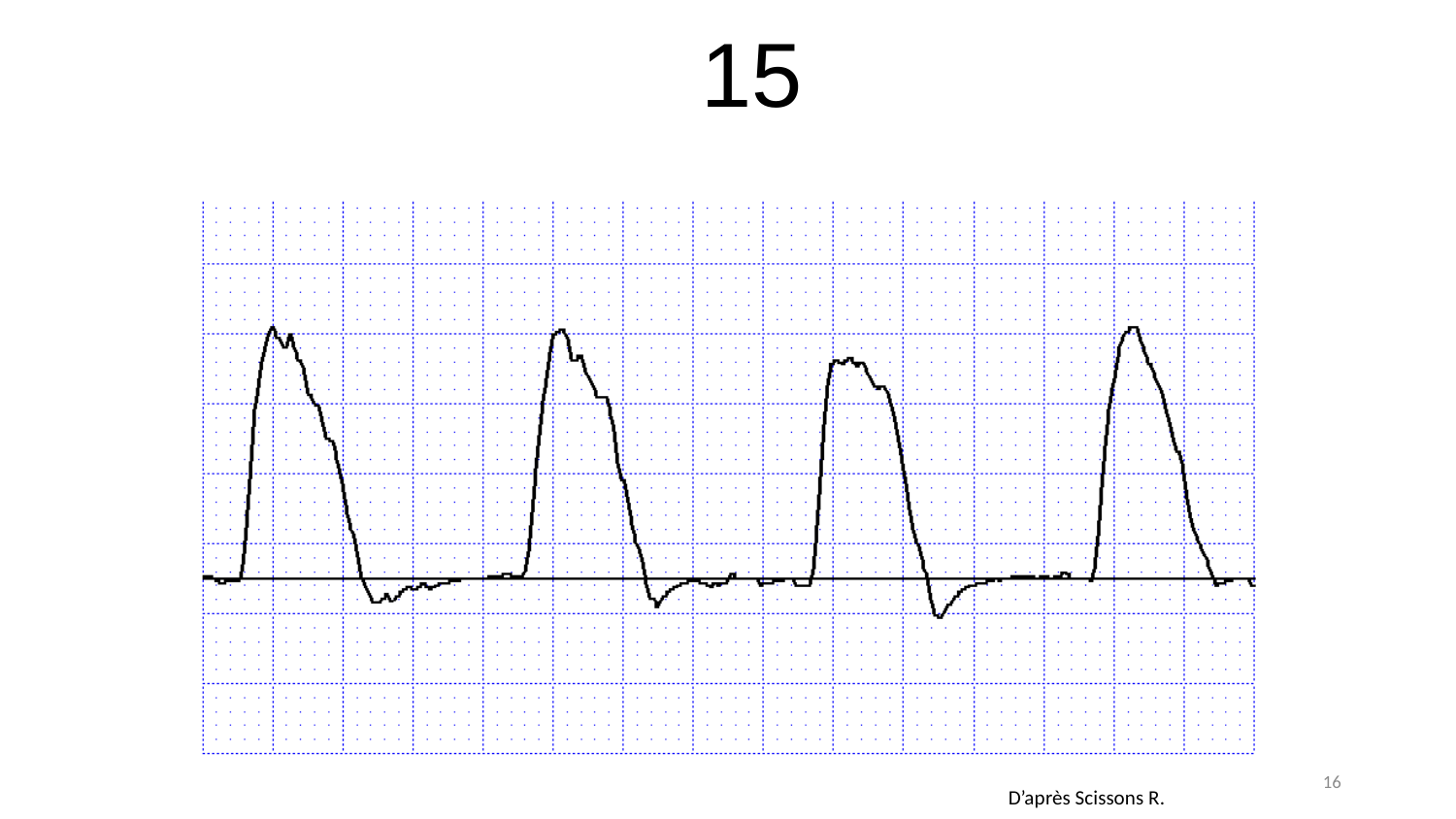

15
16
D’après Scissons R.

## Slide 17
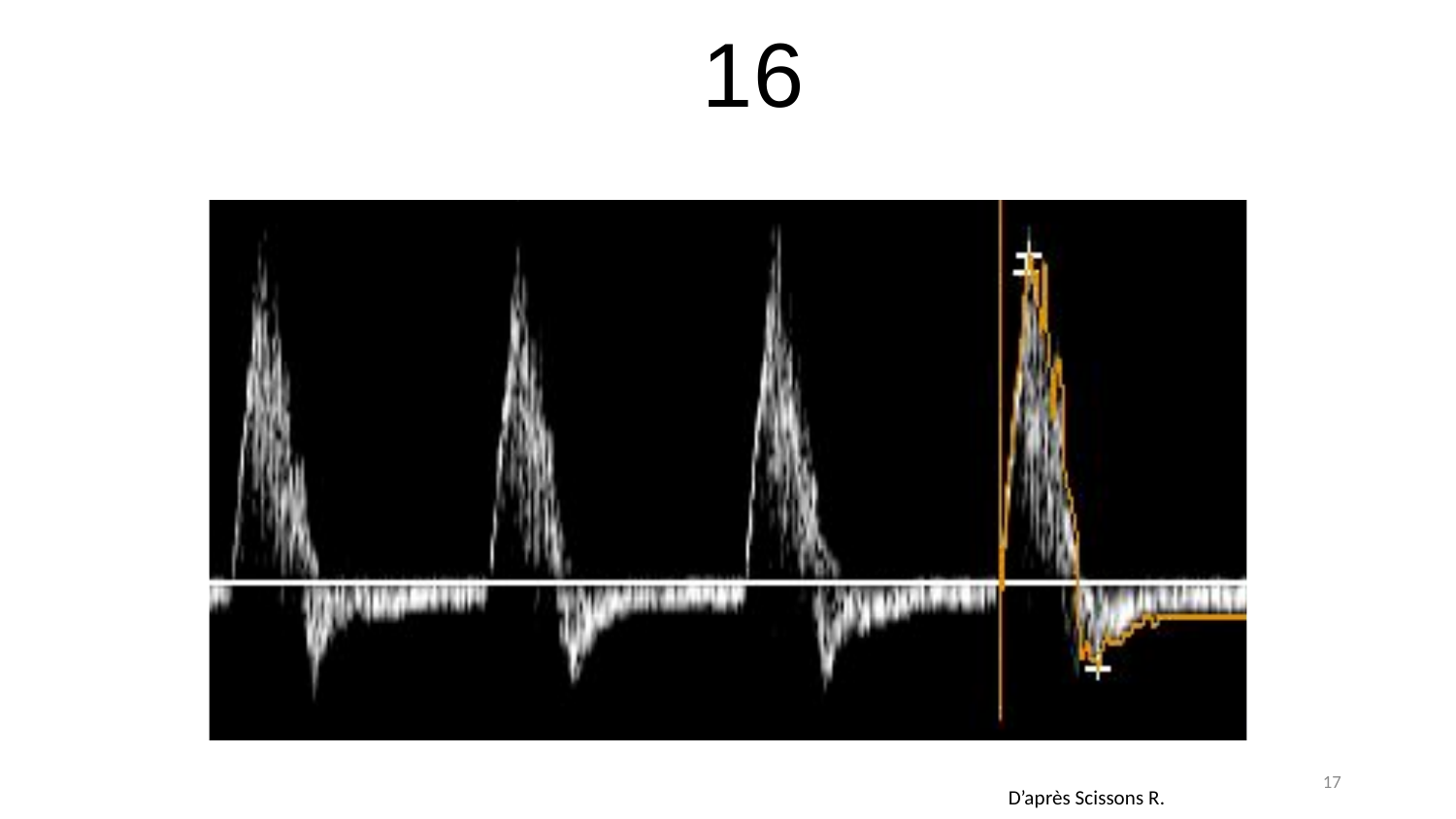

16
17
D’après Scissons R.

## Slide 18
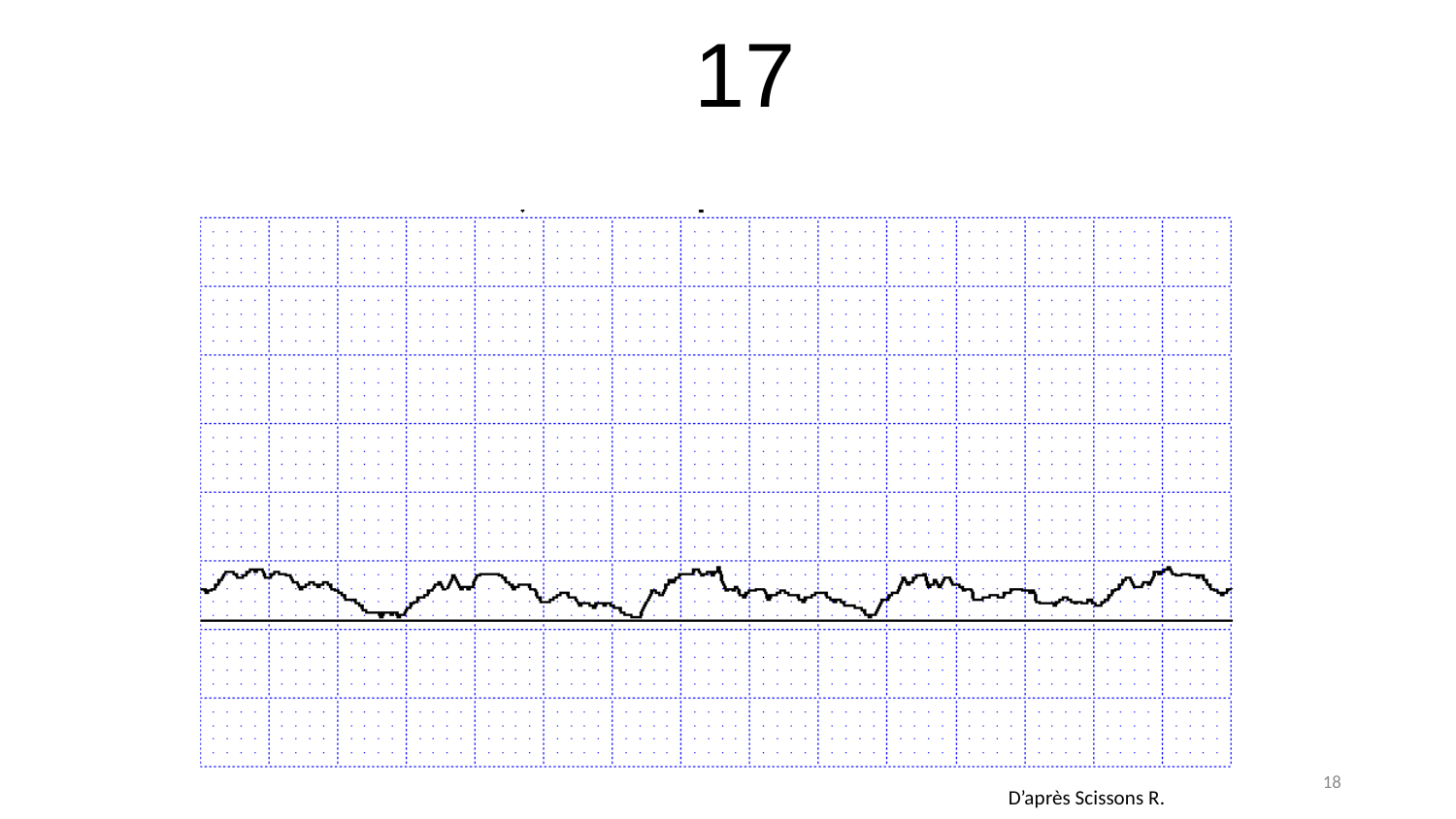

17
18
D’après Scissons R.

## Slide 19
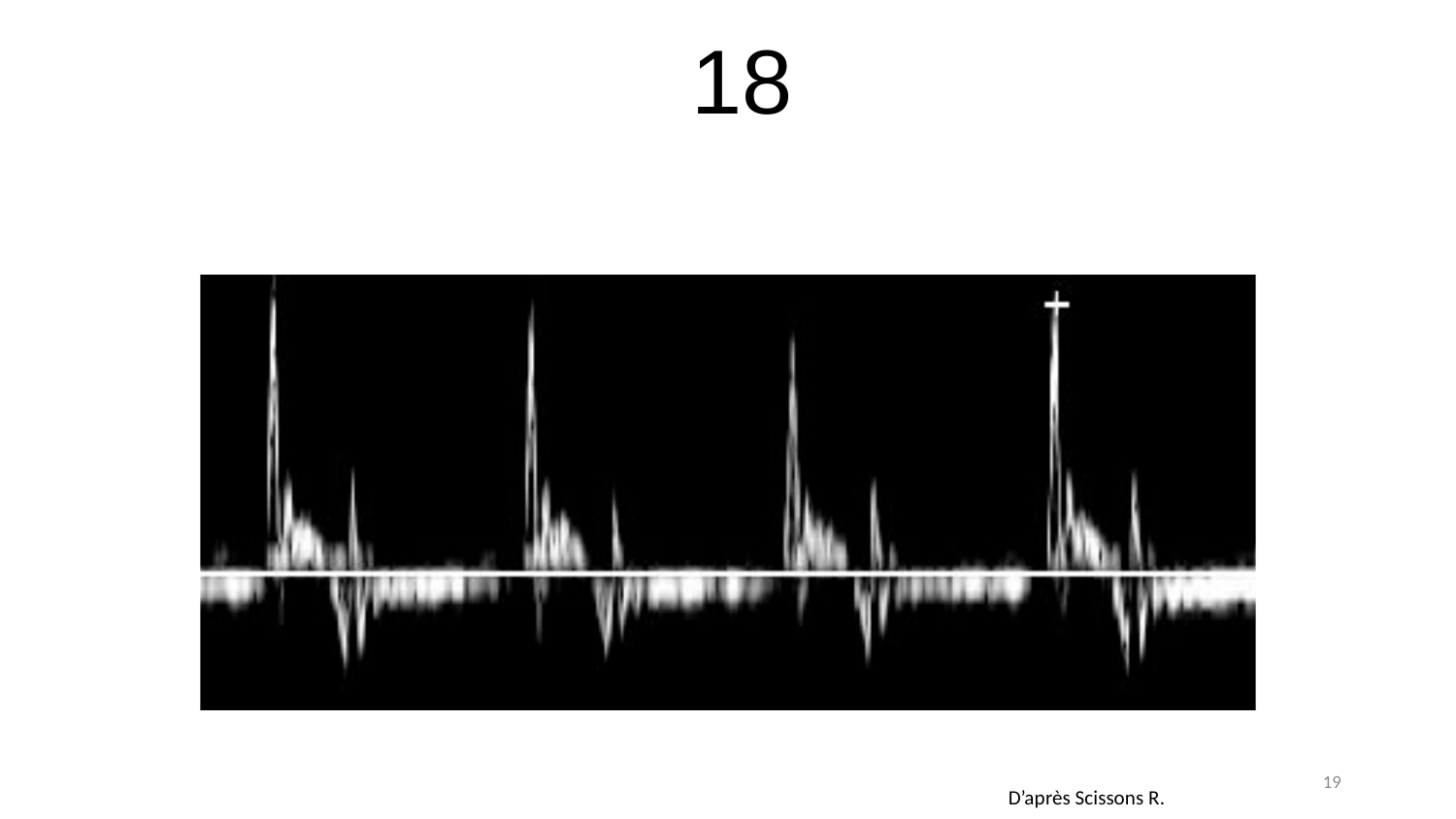

18
19
D’après Scissons R.

## Slide 20
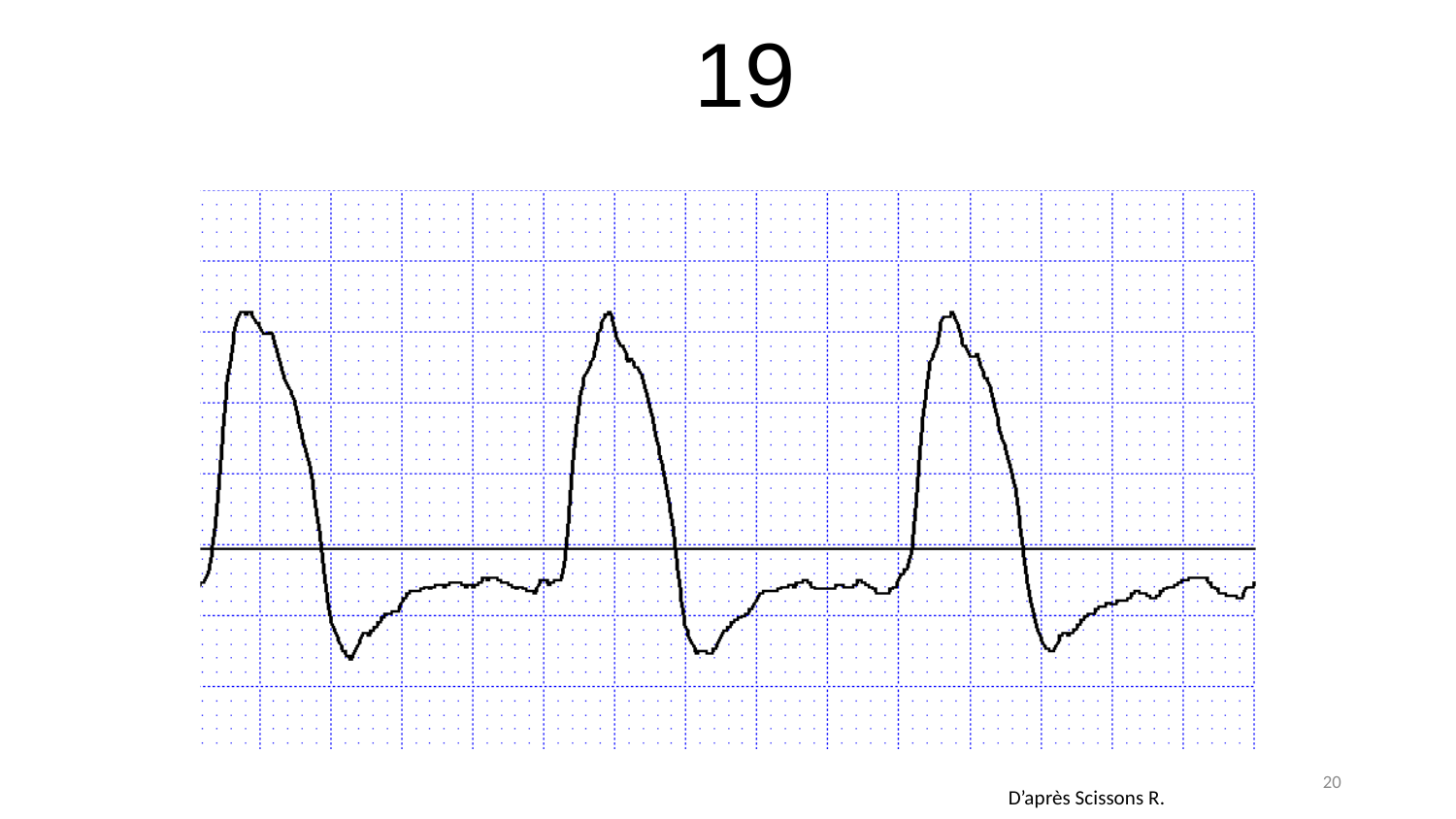

19
20
D’après Scissons R.

## Slide 21
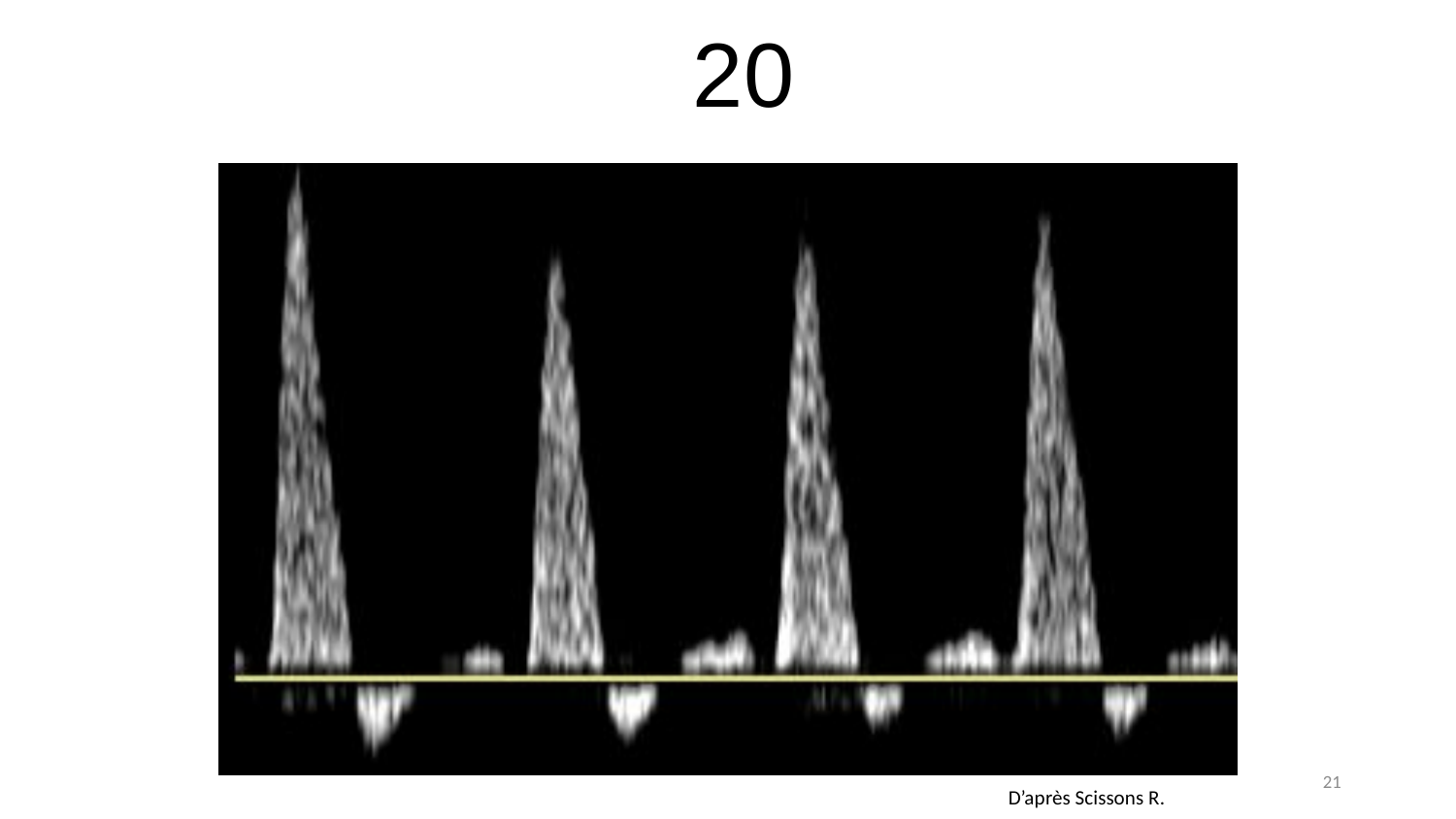

20
21
D’après Scissons R.

## Slide 22
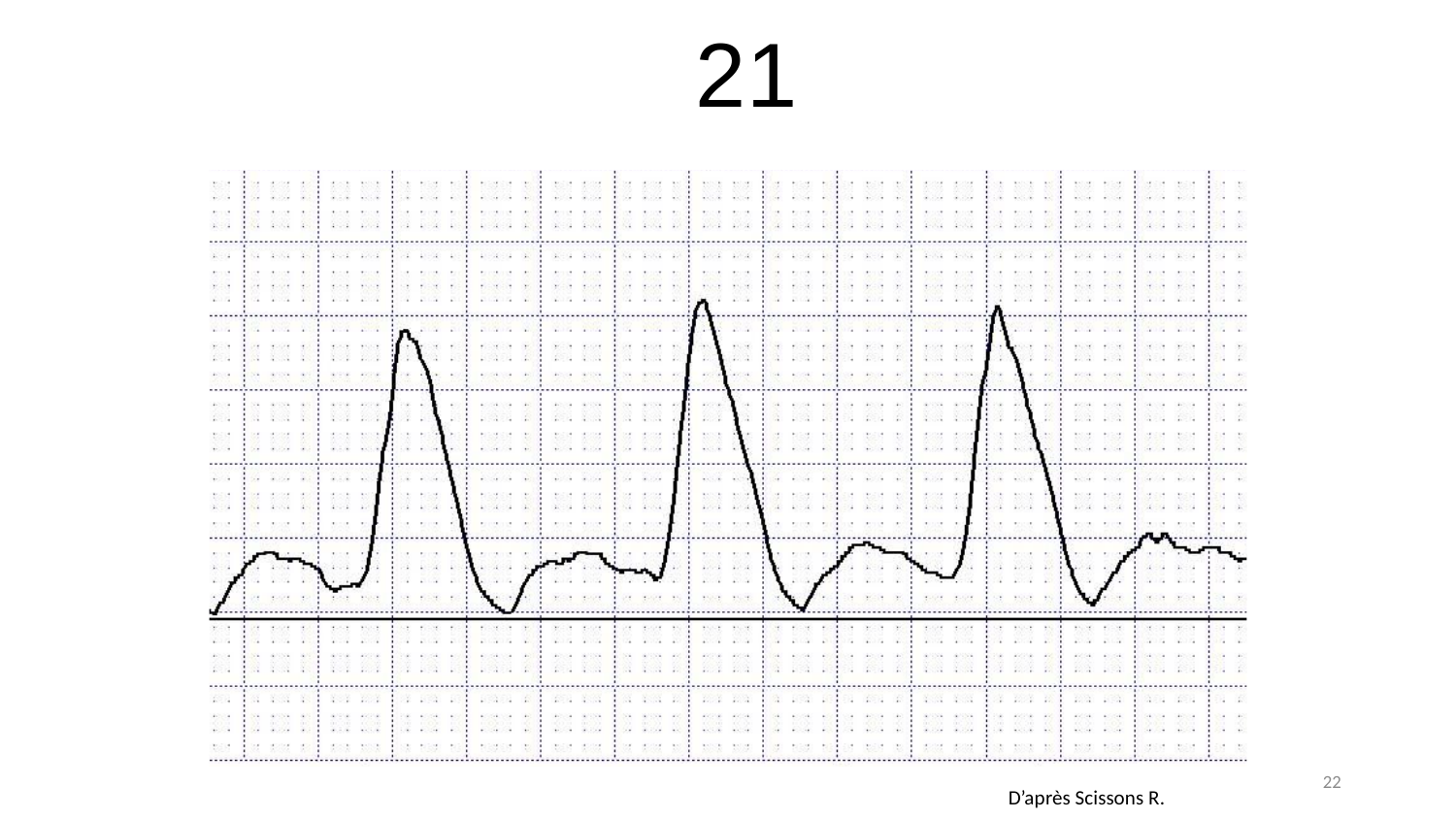

21
22
D’après Scissons R.

## Slide 23
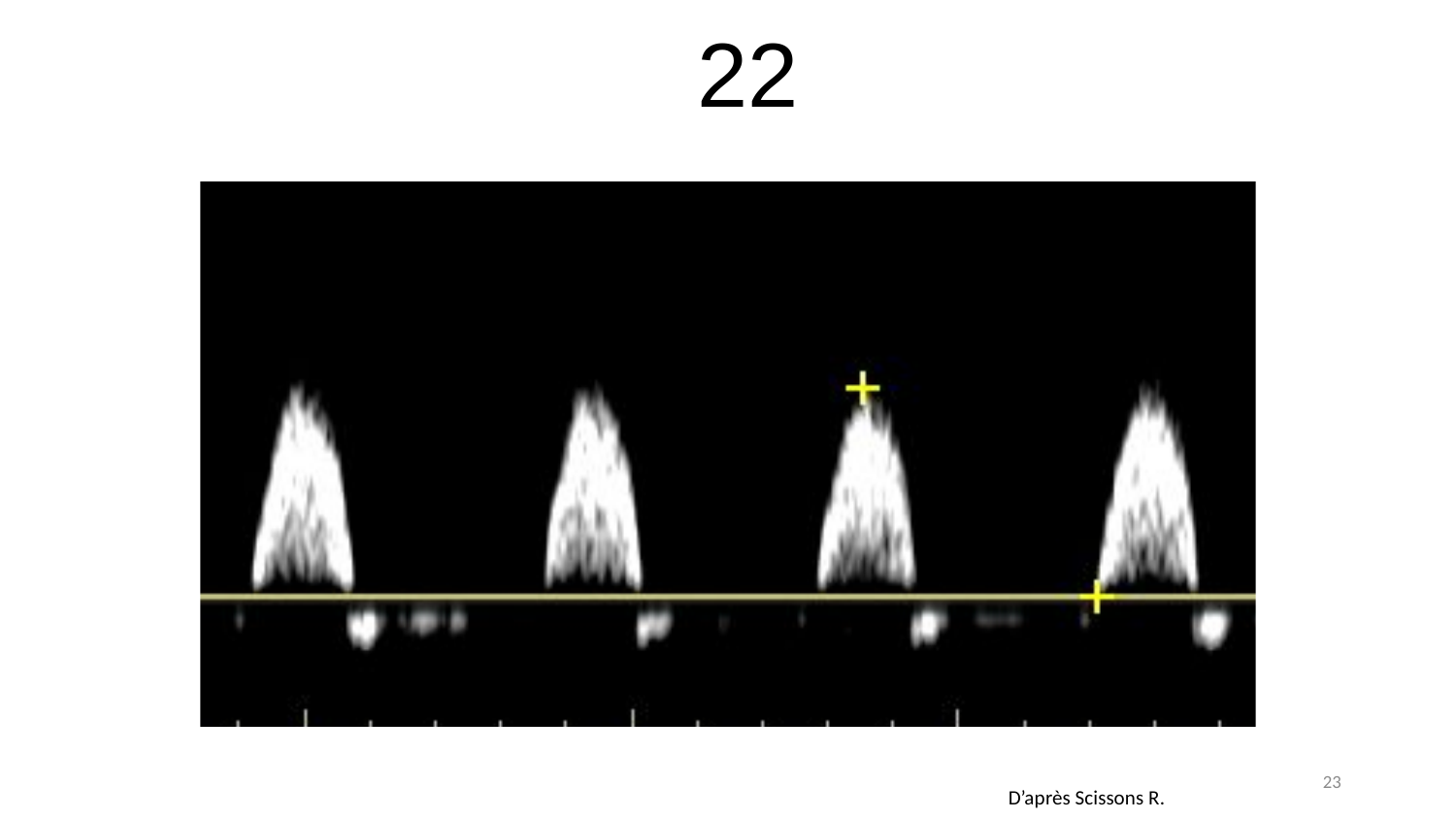

22
23
D’après Scissons R.

## Slide 24
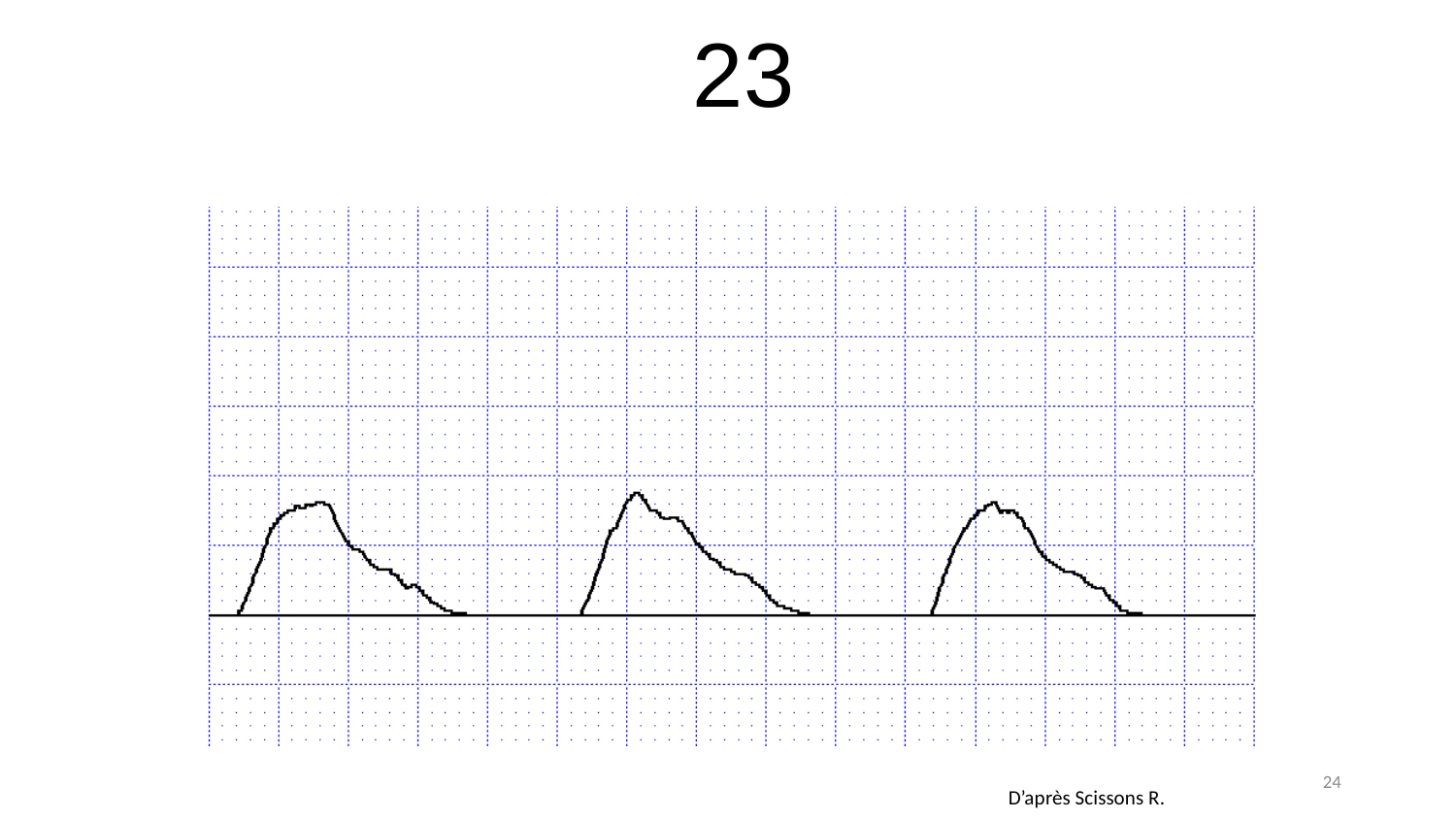

23
24
D’après Scissons R.

## Slide 25
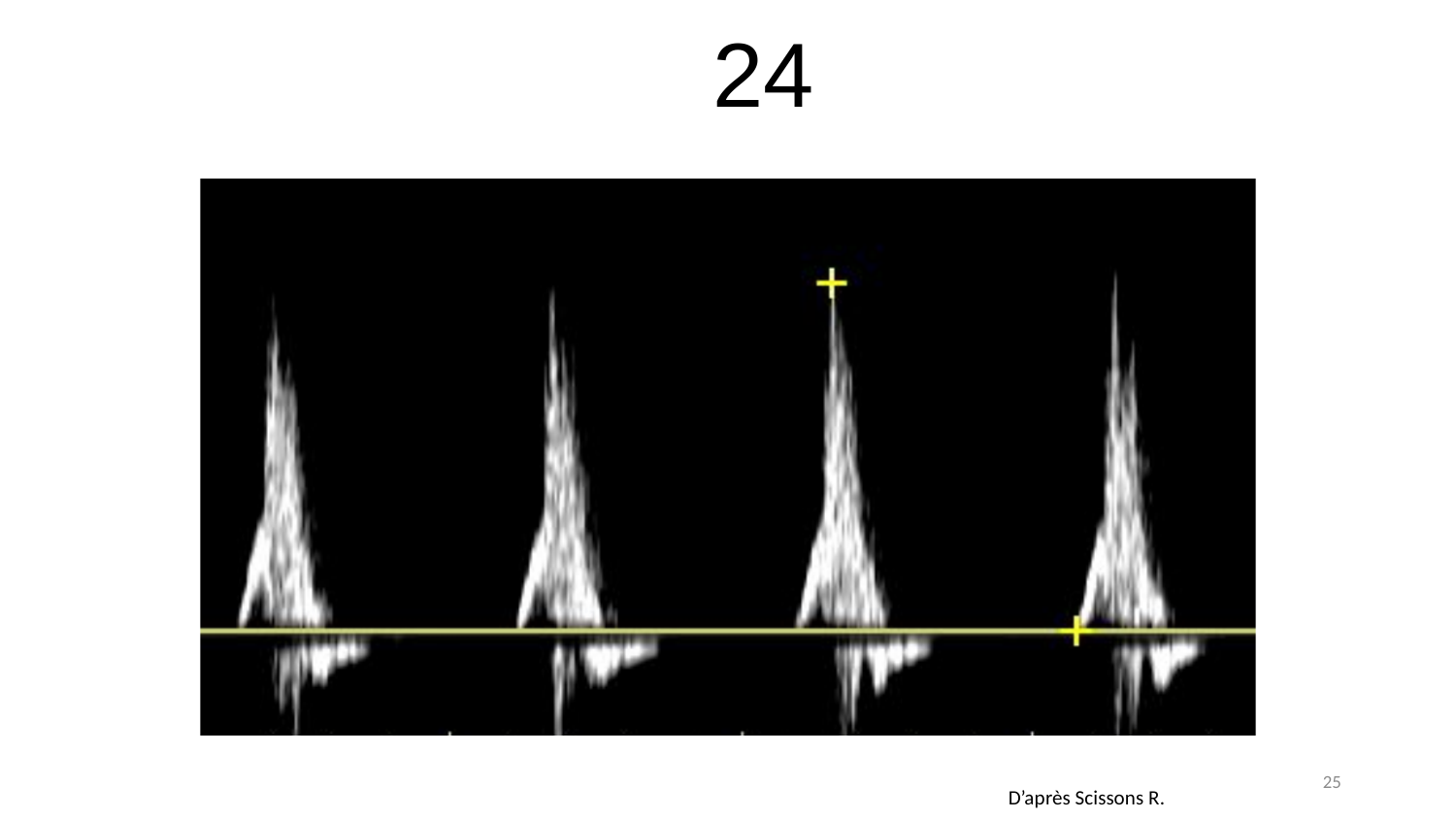

24
25
D’après Scissons R.

## Slide 26
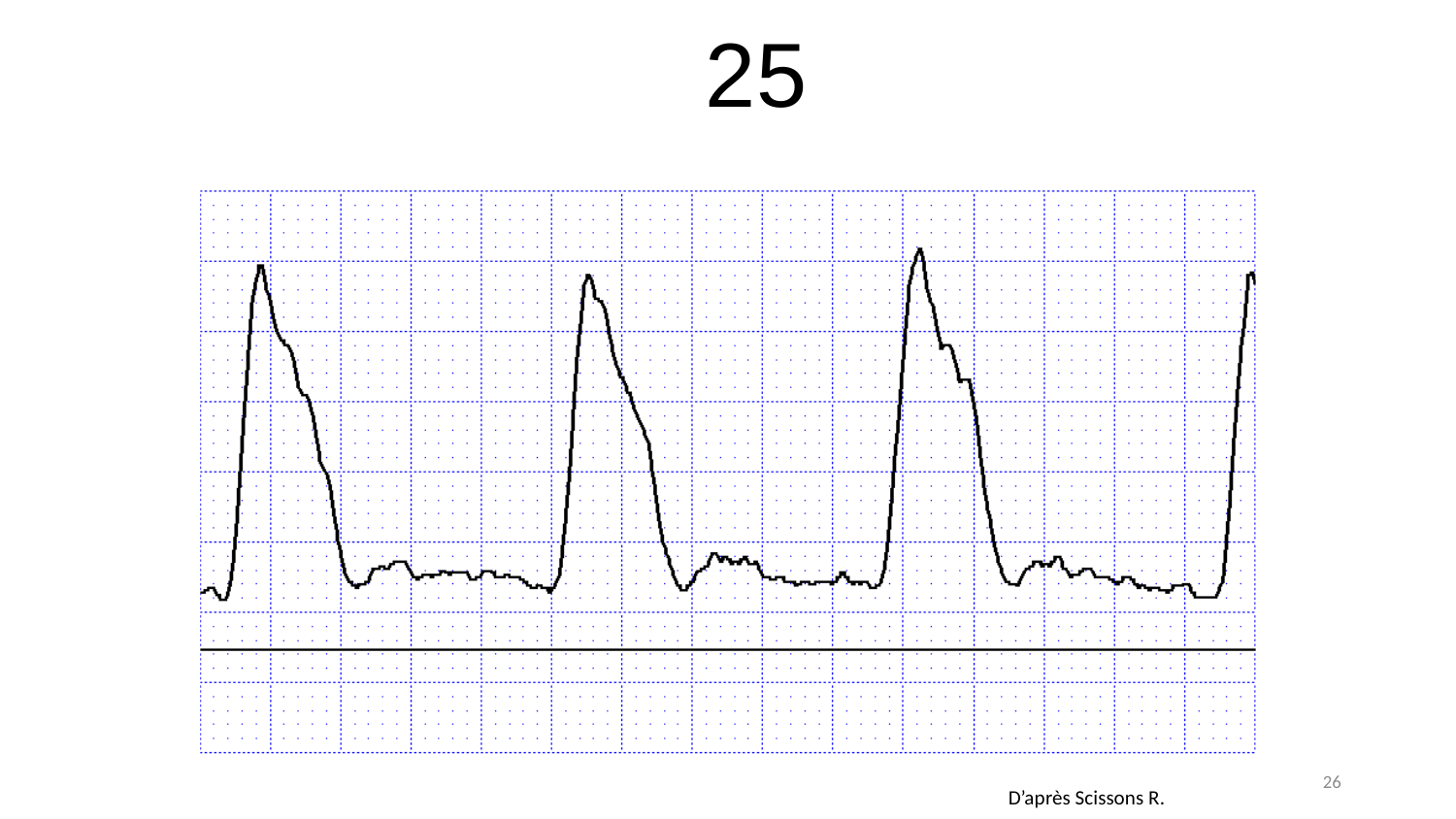

25
26
D’après Scissons R.

## Slide 27
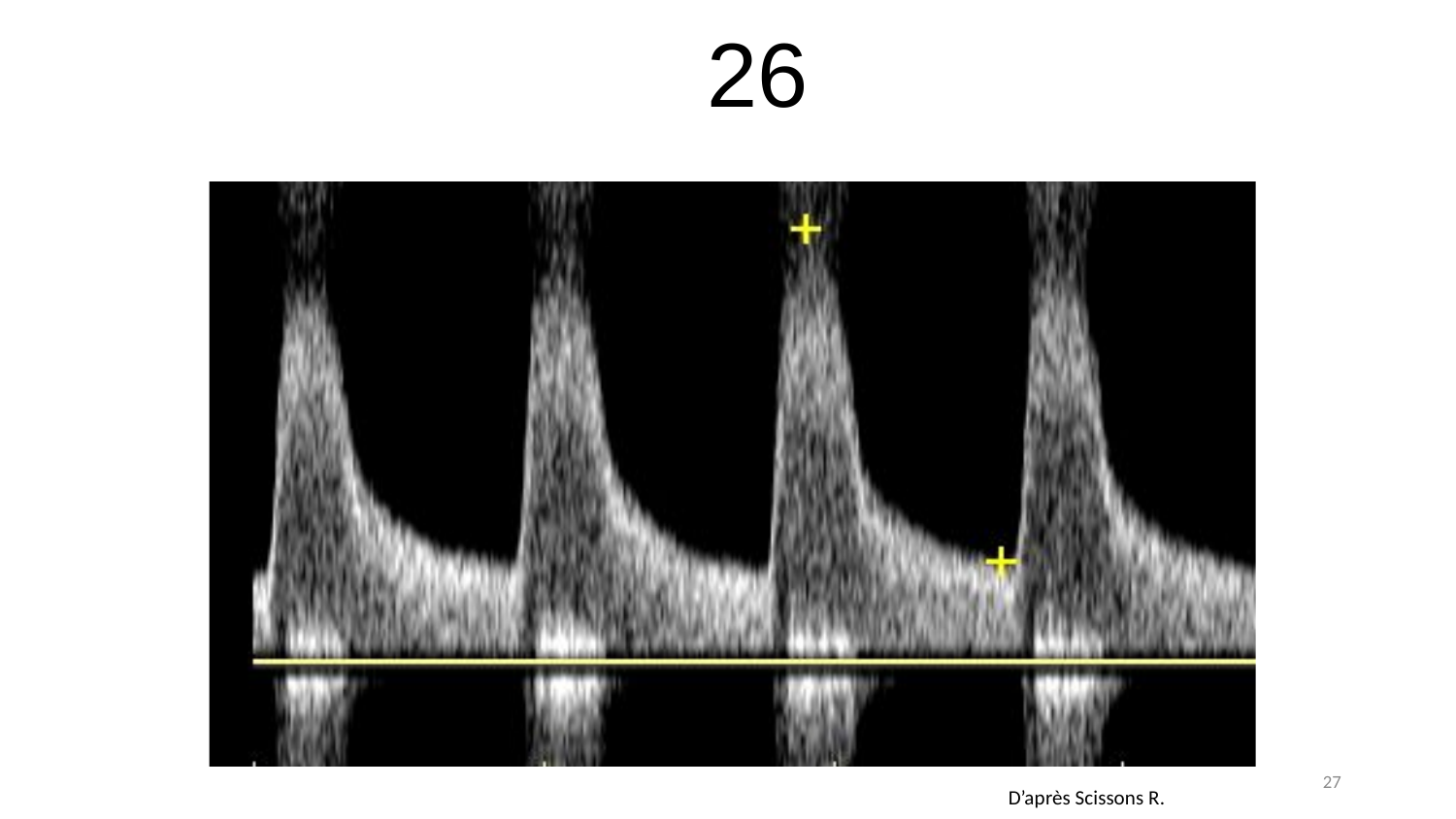

26
27
D’après Scissons R.

## Slide 28
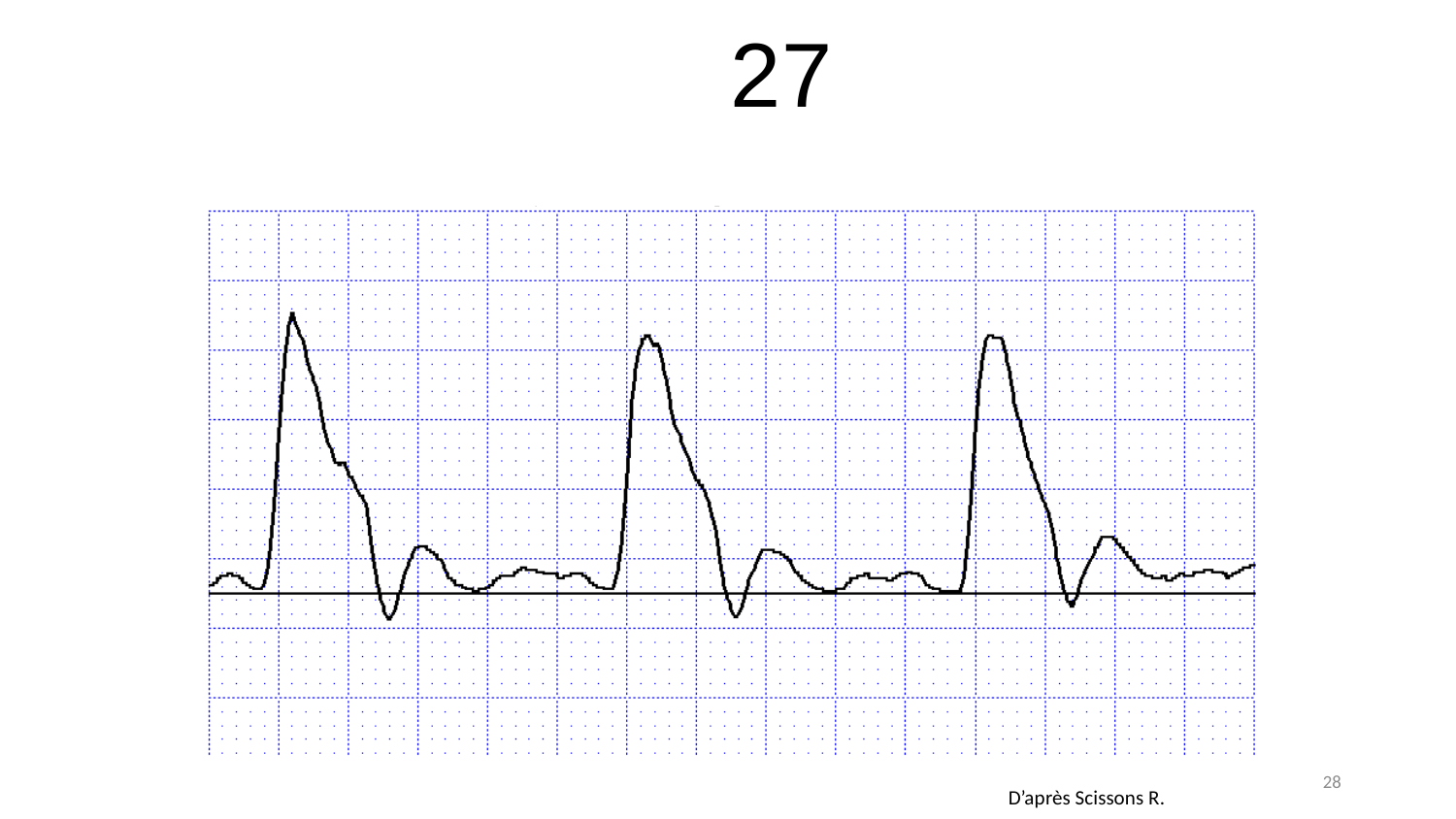

27
28
D’après Scissons R.

## Slide 29
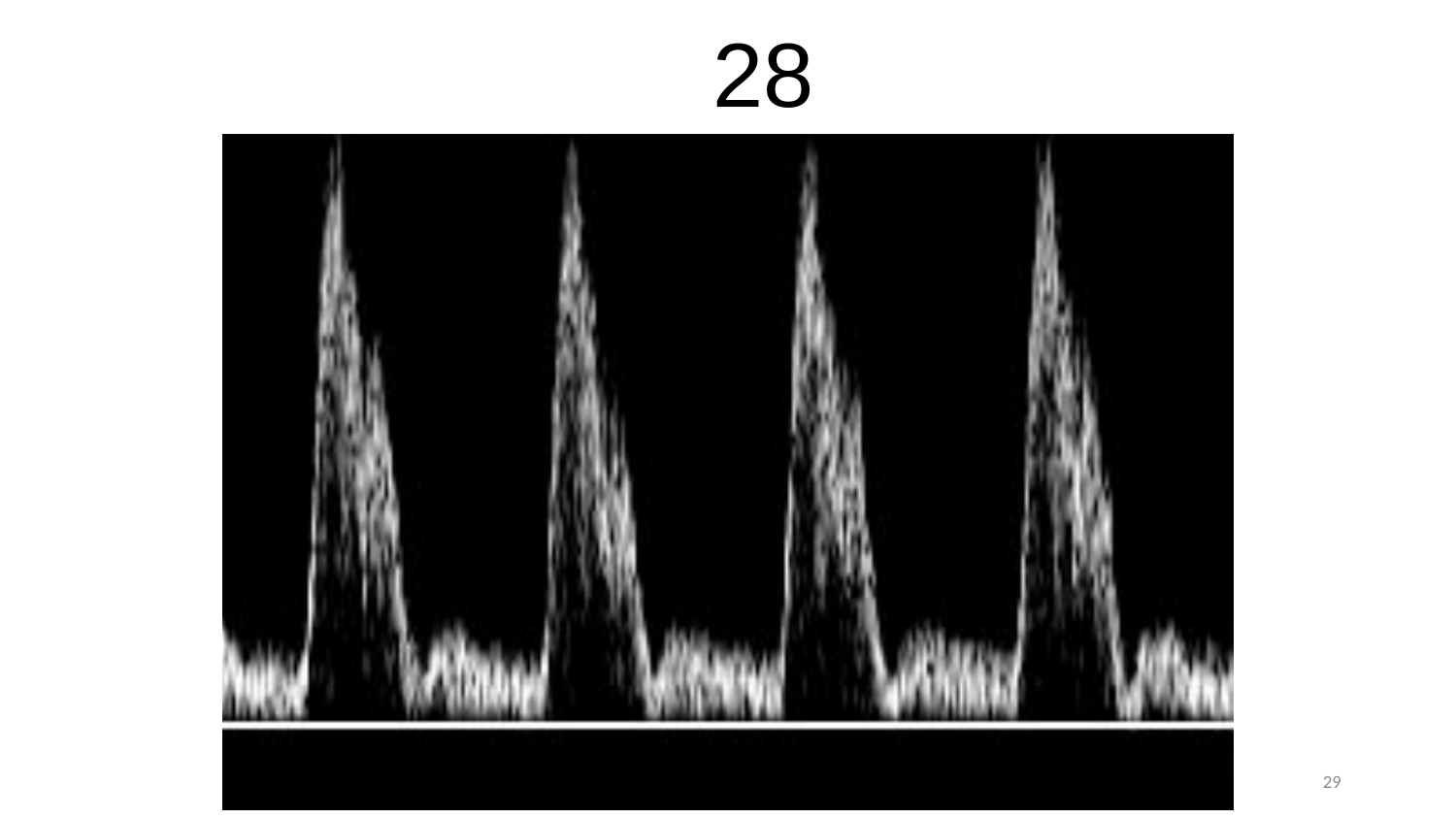

28
29
D’après Scissons R.

## Slide 30
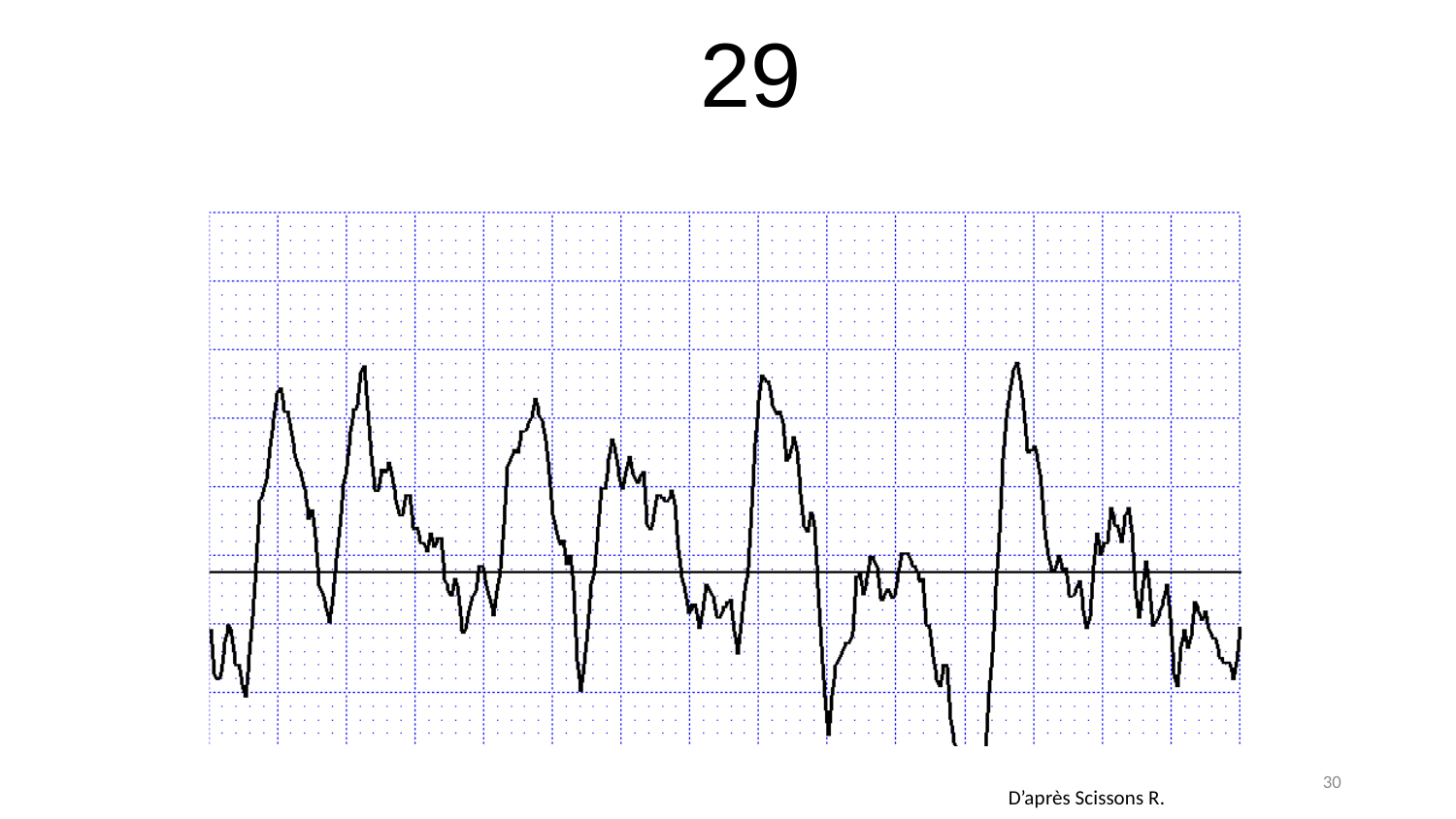

29
30
D’après Scissons R.

## Slide 31
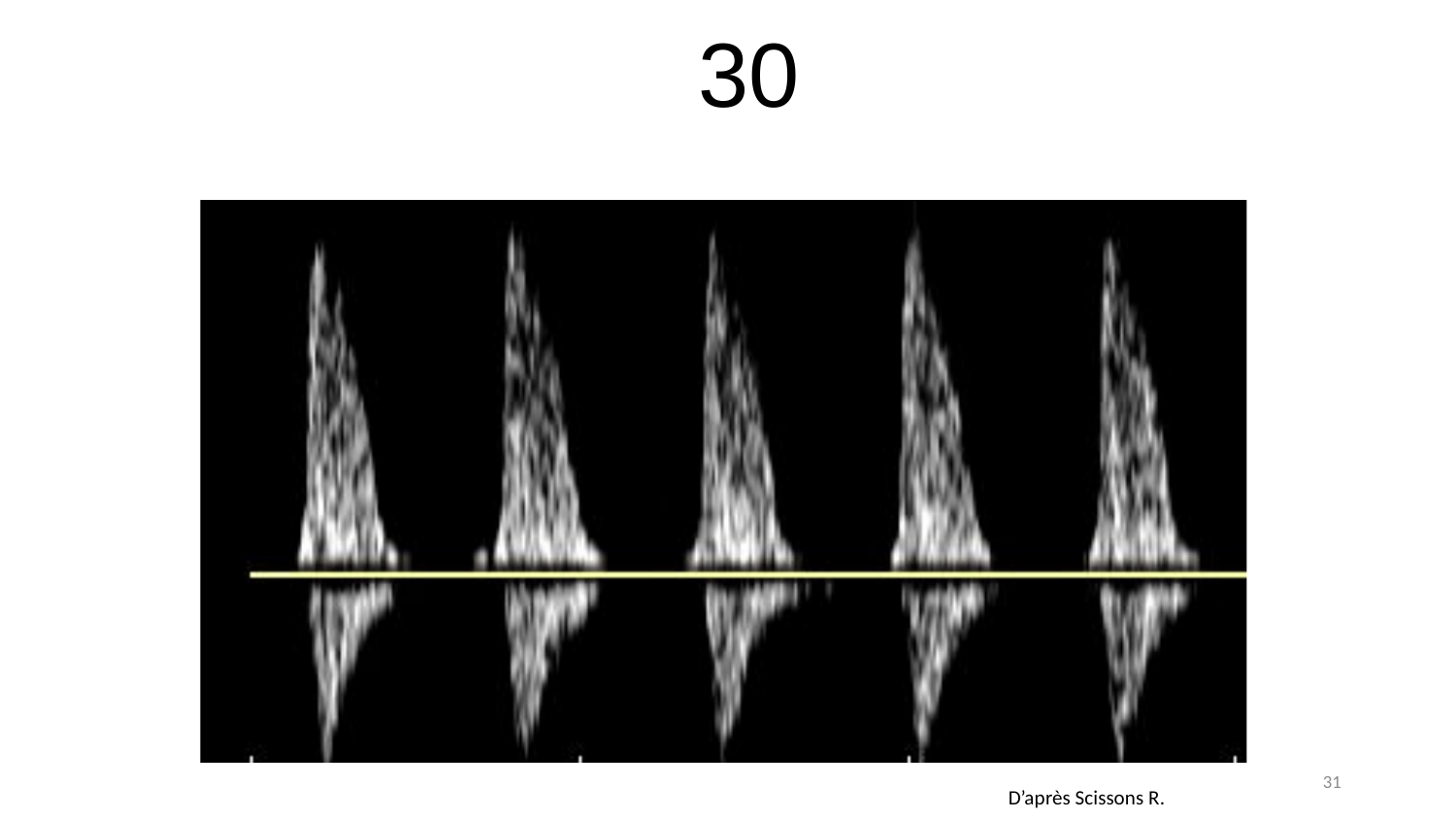

30
31
D’après Scissons R.

## Slide 32
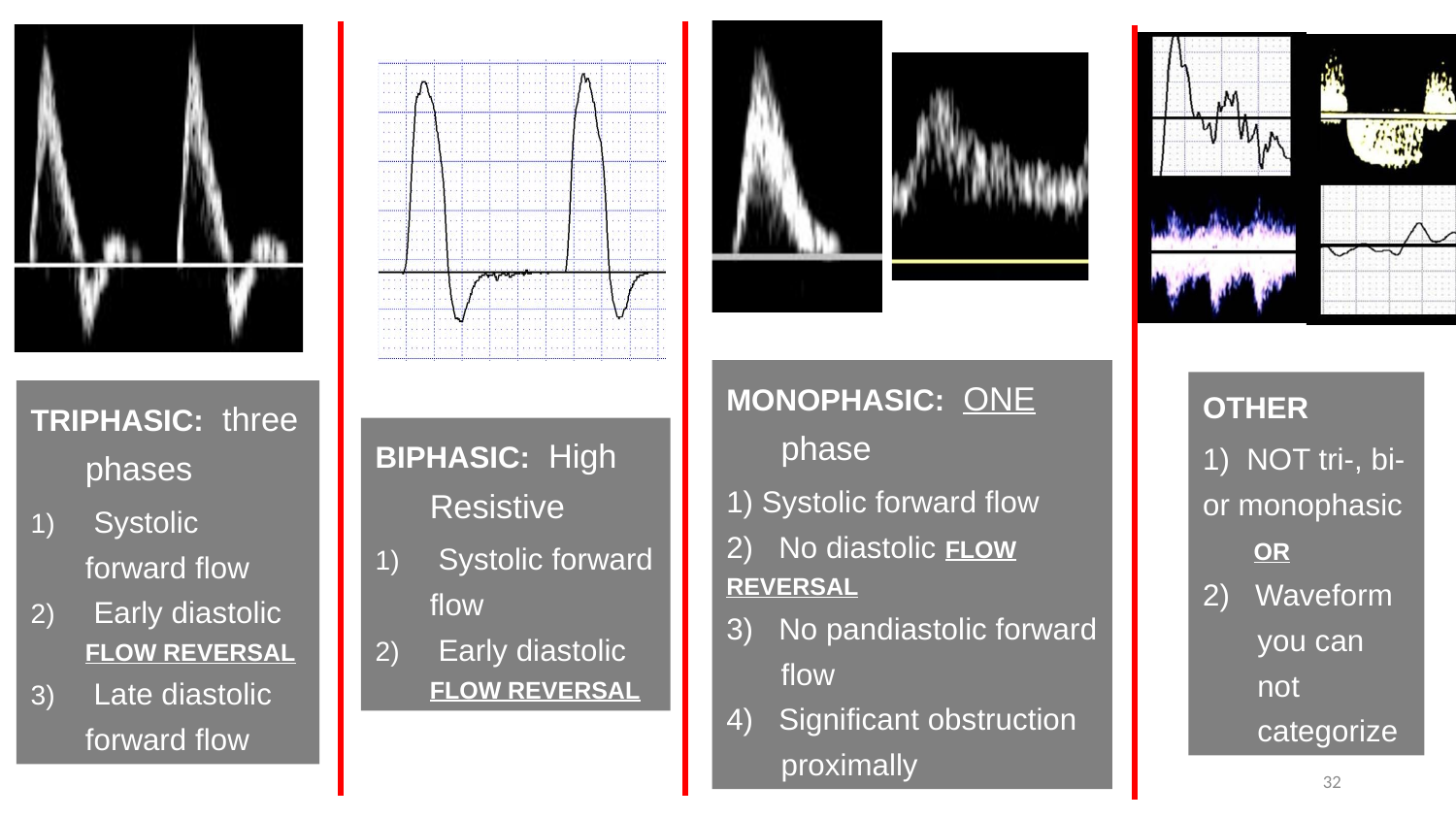

MONOPHASIC: ONE phase
1) Systolic forward flow
2) No diastolic FLOW REVERSAL
3) No pandiastolic forward flow
4) Significant obstruction proximally
OTHER
1) NOT tri-, bi- or monophasic
 OR
2) Waveform you can not categorize
TRIPHASIC: three phases
 Systolic forward flow
 Early diastolic FLOW REVERSAL
 Late diastolic forward flow
BIPHASIC: High Resistive
 Systolic forward flow
 Early diastolic FLOW REVERSAL
32
